# Heterogeneous patterns of DNA methylation-based field effects in histologically normal prostate tissue from cancer patients

**DOI:** 10.1038/srep40636

**Published:** 2017-01-13

**Authors:** Mia Møller, Siri Hundtofte Strand, Kamilla Mundbjerg, Gangning Liang, Inderbir Gill, Christa Haldrup, Michael Borre, Søren Høyer, Torben Falck Ørntoft, Karina Dalsgaard Sørensen

**Affiliations:** 1 Department of Molecular Medicine, Aarhus University Hospital, Aarhus, Denmark; 2 Keck School of Medicine of University of Southern California, Los Angeles, California, USA; 3 Department of Urology, Aarhus University Hospital, Aarhus, Denmark; 4 Department of Pathology, Aarhus University Hospital, Aarhus, Denmark

## Abstract

Prostate cancer (PC) diagnosis is based on histological evaluation of prostate needle biopsies, which have high false negative rates. Here, we investigated if cancer-associated epigenetic field effects in histologically normal prostate tissue may be used to increase sensitivity for PC. We focused on nine genes (*AOX1, CCDC181 (C1orf114*), *GABRE, GAS6, HAPLN3, KLF8, MOB3B, SLC18A2*, and *GSTP1*) known to be hypermethylated in PC. Using quantitative methylation-specific PCR, we analysed 66 malignant and 134 non-malignant tissue samples from 107 patients, who underwent ultrasound-guided prostate biopsy (67 patients had at least one cancer-positive biopsy, 40 had exclusively cancer-negative biopsies). Hypermethylation was detectable for all genes in malignant needle biopsy samples (AUC: 0.80 to 0.98), confirming previous findings in prostatectomy specimens. Furthermore, we identified a four-gene methylation signature (*AOX1*x*GSTP1*x*HAPLN3*x*SLC18A2*) that distinguished histologically non-malignant biopsies from patients with *vs*. without PC in other biopsies (AUC = 0.65; sensitivity = 30.8%; specificity = 100%). This signature was validated in an independent patient set (59 PC, 36 adjacent non-malignant, and 9 normal prostate tissue samples) analysed on Illumina 450 K methylation arrays (AUC = 0.70; sensitivity = 40.6%; specificity = 100%). Our results suggest that a novel four-gene signature may be used to increase sensitivity for PC diagnosis through detection of epigenetic field effects in histologically non-malignant prostate tissue samples.

Prostate cancer (PC) is the second leading cause of cancer in men worldwide^1^. In 2012, more than 1.1 million men were diagnosed with PC and an estimated 300,000 men died of the disease^1^. Symptoms of PC are unspecific and diagnosis is generally based on an elevated level of serum prostate-specific antigen (PSA) and/or a suspect digital rectal examination (DRE) followed by histological evaluation of prostate needle biopsies^2^. An elevated PSA level, however, is not specific for PC and there is no specific value above which PSA indicates PC^3^. Thus, up to two thirds of elevated PSA tests indicating PC are false positives (*i.e*. no cancer detected by biopsy), while on the other hand approximately 15% of men with PC do not have elevated PSA^4^,^5^. Moreover, needle biopsy has limited sensitivity as only a small volume of the prostate is sampled. Thus, prostate biopsy is associated with ~10–30% false negative rates (initial negative biopsy followed by positive repeat biopsy)^6^,^7^,^8^,^9^,^10^,^11^,^12^, which may not only cause delayed diagnosis and postponement of treatment, but is also associated with a considerable risk of sepsis for each biopsy procedure performed^13^. Accordingly, improved methods for PC diagnosis are needed to reduce the number of unnecessary prostate biopsies and ensure early detection of potentially aggressive PCs that need treatment.

Aberrant DNA promoter hypermethylation has shown promising potential as a source for PC biomarker discovery^14^,^15^. Such epigenetic alterations commonly precede genetic changes in PC development and generally display more consistent patterns between tumours than genetic aberrations^16^. To date, several genes have been identified as common targets for aberrant promoter hypermethylation in PC^17^, including the extensively studied *GSTP1* (Glutathione S-Transferase pi 1) gene that is hypermethylated in more than 90% of all PC tissue samples^18^,^19^. Moreover, we have previously reported similarly high frequencies of cancer-specific promoter hypermethylation for the eight biomarker candidate genes *AOX1* (Aldehyde Oxidase 1), *CCDC181* (Coiled-Coil Domain Containing 181, also known as *C1orf114*), *GABRE* (Gamma-Aminobutyric Acid A Receptor Epsilon), *GAS6* (Growth Arrest-Specific 6), *HAPLN3* (Hyaluronan and Proteoglycan Link Protein 3), *KLF8* (Kruppel-like Factor 8), *MOB3B* (MOB kinase activator 3B), and *SLC18A2* (Solute Carrier Family 18 vesicular monoamine Member 2) in malignant tissue samples from radical prostatectomy (RP) specimens^20^,^21^,^22^,^23^. However, while hypermethylation-based cancer field effects have been demonstrated for *GSTP1* in several previous studies of PC^24^,^25^,^26^,^27^,^28^,^29^, the existence of such epigenetic field effects remains to be investigated for our eight novel candidate methylation marker genes.

Detection of cancer field effects in histologically normal prostate tissue adjacent to PC could potentially be used to increase the diagnostic sensitivity and/or guide the need for repeat biopsy. So far, field effects in relation to PC have been reported at various molecular levels, including RNA^30^,^31^, DNA^32^, protein^33^,^34^, and DNA methylation^35^,^36^,^37^,^38^, where the latter seems particularly promising. Indeed, a commercial test (ConfirmMDx for Prostate Cancer, MDx Health), based on *APC* (Adenomatous Polyposis Coli), *GSTP1*, and *RASSF1* (Ras Association (RalGDS/AF-6) Domain Family Member 1) hypermethylation in cancer-negative biopsies, offers a negative predictive value of 90%^39^. Moreover, results from several previous studies suggest that detection of hypermethylated *GSTP1* and *APC* in cancer-negative prostate biopsies – either only these two genes^26^ or in combination with *RARB2* (Retinoic Acid Receptor, beta transcript 2)^25^ or *RASSF1*^28^,^29^ - may also hold potential to increase diagnostic sensitivity by predicting a positive repeat biopsy.

In this study, we show that PC-specific hypermethylation of *AOX1, CCDC181, GABRE, GAS6, HAPLN3, KLF8, MOB3B, SLC18A2*, and *GSTP1* can be detected by qMSP even in scarce prostate tissue samples from diagnostic needle biopsies. Hence, our results confirm and expand on previous reports of PC-specific hypermethylation of these genes in prostatectomy specimens^18^,^20^,^21^,^22^,^23^. Furthermore, to investigate if epigenetic cancer field effects exist for our eight novel candidate genes, we analysed non-malignant diagnostic needle biopsy samples from 79 patients with/without cancer in other biopsies using qMSP. We observed heterogeneous patterns of methylation-based epigenetic field effects and identified a novel four-gene field effect signature (*AOX1*x*GSTP1*x*HAPLN3*x*SLC18A2*) that was specifically associated with PC (30.8% sensitivity at 100% fixed specificity). This four-gene signature was successfully validated using Illumina 450 K methylation array data from an independent patient set (40.6% sensitivity for PC at 100% fixed specificity). Notably, the diagnostic accuracy of this signature was not simply driven by *GSTP1*, for which epigenetic cancer field effects have previously been demonstrated in PC. To the best of our knowledge, this is the first study to demonstrate significant epigenetic field effects for *AOX1, HAPLN3*, and *SLC18A2* in PC.

## Results

### Detection of PC-specific hypermethylation in needle biopsy samples

By analysis of RP specimens, we have previously identified the eight genes *AOX1, CCDC181 (C1orf114*), *GABRE, GAS6, HAPLN3, KLF8, MOB3B*, and *SLC18A2* as new common targets of aberrant promoter hypermethylation in PC^20^,^21^,^22^. Here, we initially tested if cancer-specific hypermethylation of these genes can be detected also in routinely processed sections of diagnostic prostate needle biopsies, where only limited amounts of FFPE tissue are available for DNA extraction and molecular analysis. For comparison, we included *GSTP1*, which is the most extensively studied candidate methylation marker for PC to date^40^.

The methylation level of each gene was analysed by qMSP in prostate needle biopsy samples from a total of 107 patients who underwent TRUS-guided prostate biopsy due to suspicion of PC. Out of 107 patients examined, 67 had at least one cancer positive biopsy, whereas the remaining 40 patients had exclusively cancer-negative biopsies. Based on histopathological diagnostic examination, prostate biopsy cores were divided into three sample subtypes: malignant (i.e. biopsies with histologically confirmed PC), non-malignant (NM; histologically non-malignant biopsies from patients with exclusively cancer-negative biopsies), and adjacent normal samples (AN; histologically normal biopsies from patients with PC in at least one other biopsy). Thus, the final biopsy set used for qMSP analysis included malignant samples from 48 patients, NM samples from 40 patients, and AN samples from 39 patients (Table 1; For further details, see Methods and Suppl. Fig. S1).

**Figure 1:**
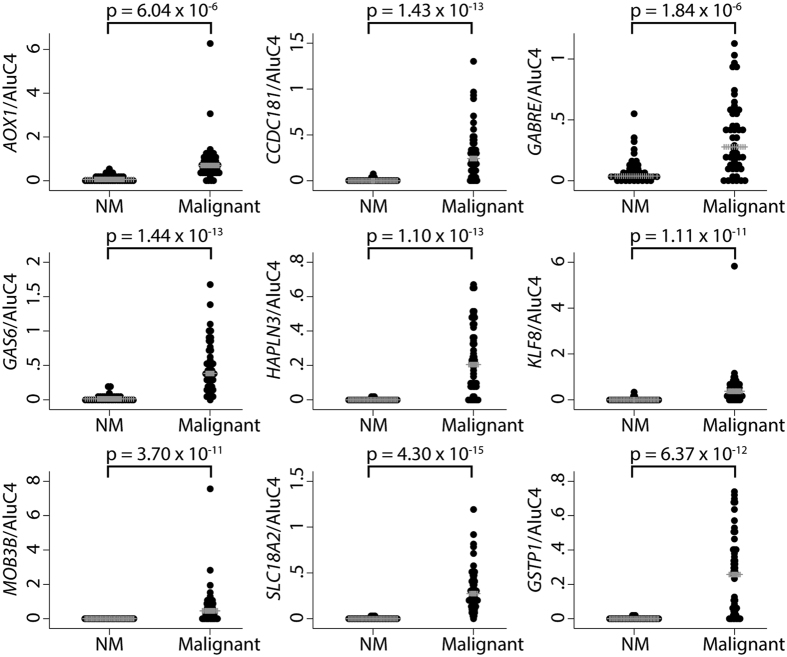
Methylation levels in malignant biopsy samples (n = 48) compared to non-malignant biopsy samples (NM, n = 40), as determined by qMSP. Grey lines indicate median methylation status within each group. Statistically significant hypermethylation in cancer samples was observed for all genes (p < 0.05 in Mann-Whitney U test). Normalisation to AluC4 was performed for all genes.

**Figure 2:**
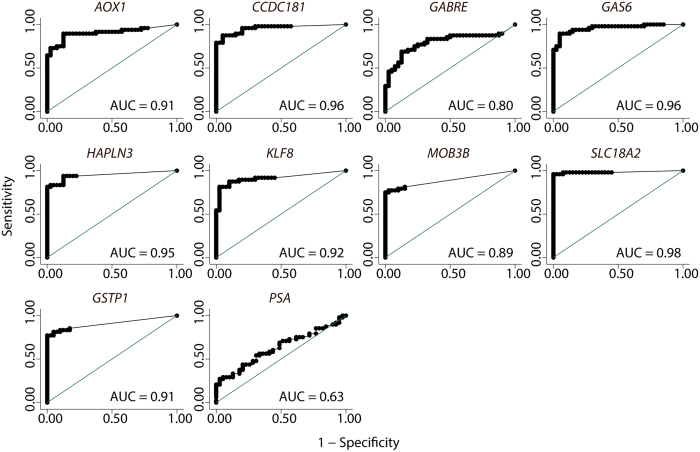
Receiver operating characteristics (ROC) curves illustrating the differences in methylation levels observed between malignant (n = 48) and non-malignant samples (n = 40) in prostate needle biopsies. All methylation assays performed better than PSA in this patient sample set. The diagonal line corresponds to no discrimination between groups.

We found that all eight candidate genes, as well as *GSTP1*, were significantly (p < 0.00002; Mann Whitney U test corrected for multiple testing) hypermethylated in malignant as compared to NM prostate biopsy samples (Fig. 1). Receiver operating characteristic (ROC) curve analysis showed high discriminative power for PC for all genes with AUCs ranging from 0.79 (*GABRE*) to 0.98 (*SLC18A2*) (Fig. 2), consistent with our previous results from RP specimens^20^,^21^,^22^. In contrast, PSA had limited diagnostic accuracy (AUC = 0.63) in this sample set (Fig. 2). When specificity was fixed at 100%, the sensitivity for PC in the biopsy sample set was 95.8% (*SLC18A2*), 81.6% (*HAPLN3*), 79.2% (*CCDC181*), 77.1% (*GSTP1*), 75.0% (*MOB3B*), 70.8% (*GAS6*), 64.6% (*AOX1*), 54.2% (*KLF8*), 29.2% (*GABRE*), and 20.8% for PSA.

**Figure 3:**
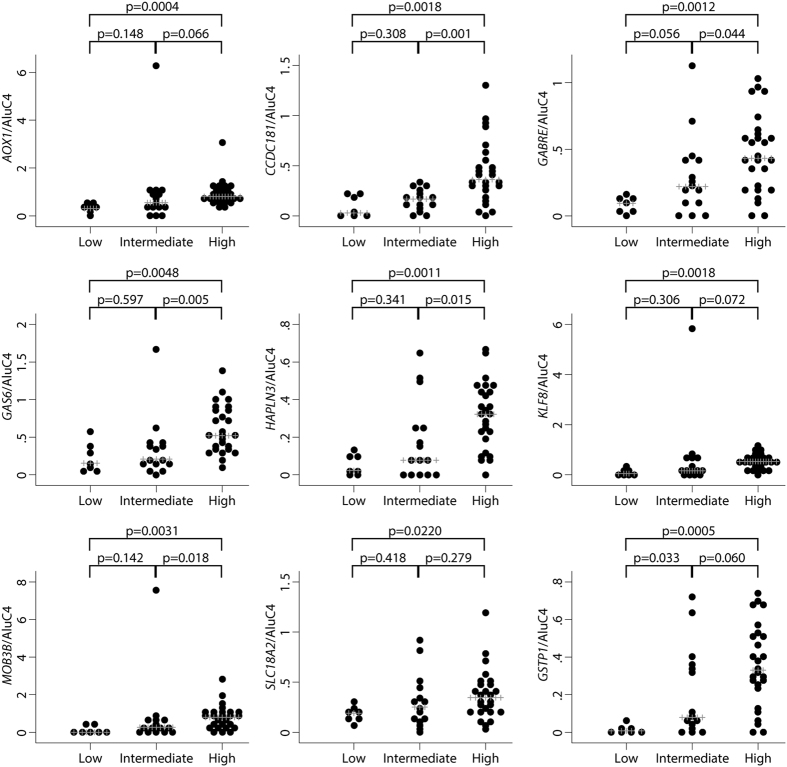
Methylation levels in malignant biopsy tissue samples in relation to PC aggressiveness defined by the D’Amico risk classification system^41^. Low risk (n = 7): PSA ≤ 10 ng/mL and Gleason score ≤6 and cT1c/cT2a; Intermediate risk (n = 15): PSA > 10 to 20 ng/mL and/or Gleason score 7 and/or cT2b; High risk (n = 26): PSA > 20 ng/mL and/or Gleason score 8–10 and/or cT2c-cT4. Higher methylation levels were significantly associated with higher risk score for all genes (p < 0.05 in Mann-Whitney U test). Normalisation to AluC4 was performed for all genes.

Next, we investigated if methylation levels in malignant biopsy samples were associated with PC aggressiveness as defined by the D’Amico risk score^41^. The D’Amico risk nomogram is based on serum PSA, Gleason score in biopsies, and cT stage, and is used for risk stratification at the time of diagnosis in order to guide treatment decisions^42^. For all nine genes, we found significantly higher methylation levels in malignant biopsy samples from high risk as compared to low risk patients (Fig. 3). Additional studies are needed to assess the potential prognostic value of our eight candidate methylation markers in diagnostic prostate biopsies; however, this is beyond the scope of the present study.

In summary, these results demonstrate that scarce amounts of FFPE prostate needle biopsy tissue, in this case leftover sections after routine histological examination, are sufficient for qMSP analysis of several candidate methylation marker genes. This further indicates that a future qMSP-based molecular diagnostic test may be developed as a relatively simple supplement to routine histological evaluation without the need for additional biopsies.

### Cancer field effects in histologically normal prostate biopsies

**Figure 4:**
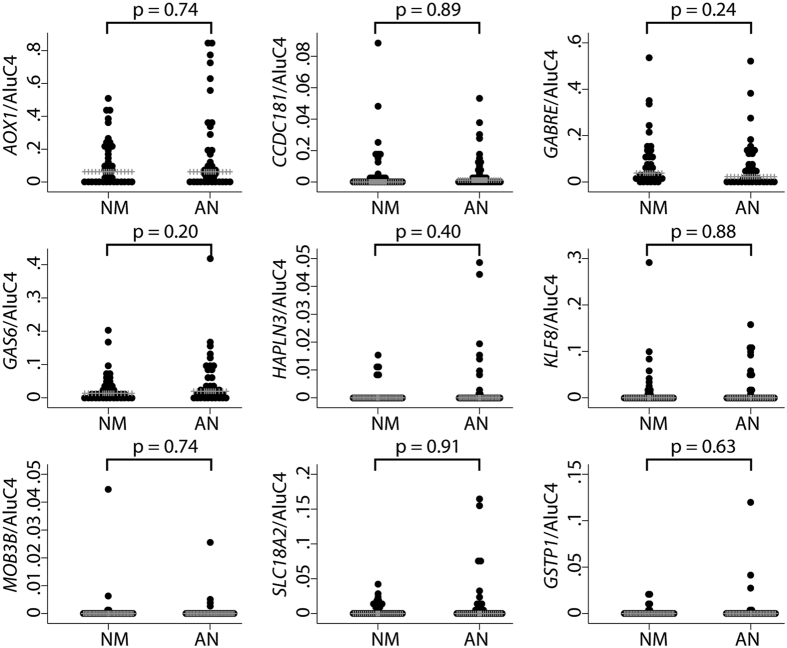
Methylation levels in adjacent normal (AN, n = 39) compared to non-malignant (NM, n = 40) tissue samples from prostate needle biopsies. Grey lines indicate median methylation levels as determined by qMSP. No statistically significant difference was observed for any of the genes (p > 0.05 in Mann-Whitney U test). Normalisation to AluC4 was performed for all genes.

Methylation-based cancer field effects have previously been reported for *GSTP1* in histologically normal prostate tissue samples from patients with PC^25^,^26^,^28^. The possible existence of such cancer field effects, however, remains to be investigated for *AOX1, CCDC181 (C1orf114*), *GABRE, GAS6, HAPLN3, KLF8, MOB3B*, and *SLC18A2*. To address this question, we performed qMSP analyses for all nine genes in histologically normal prostate biopsy samples from patients with cancer in other biopsies (AN, n = 39) vs. patients with exclusively cancer-negative biopsies (NM, n = 40). Although no significant differences in median methylation levels were seen between AN and NM biopsies for any of the nine genes tested (Fig. 4), a few highly methylated outliers were detected specifically in AN samples for *AOX1, GAS6, HAPLN3, SLC18A2*, and *GSTP1* (Fig. 4), potentially reflecting cancer field effects.

Because these highly methylated outliers were relatively rare for each single gene, we tested if multi-gene methylation signatures might increase the sensitivity for detection of PC based on epigenetic field effects. For each gene, methylation levels were dichotomised at a cut-off that ensured 100% specificity for AN vs. NM samples. Then, all nine genes were combined into every possible two-gene model (n = 36 models in total) and samples scored as hypermethylated, if at least one of the genes in the model had a methylation level above this cut-off. The five two-gene models with the lowest p-values in χ^2^ test for distinguishing AN vs. NM samples encompassed four genes: *AOX1, HAPLN3, SLC18A2*, and *GSTP1* (Suppl. Table S1), hence, these were combined into a single four-gene model.

**Figure 5:**
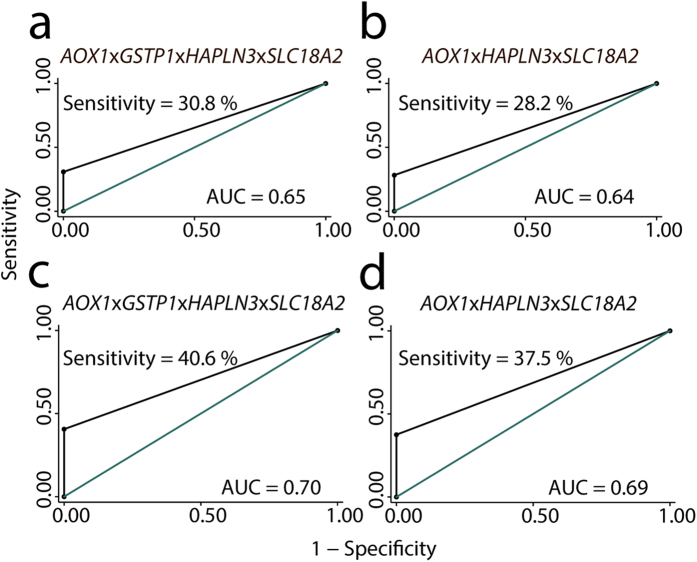
Diagnostic potential of novel epigenetic field effect signatures. (**a**,**b**) Receiver operating characteristic curves for adjacent normal (n = 39) vs. non-malignant (n = 40) tissue samples from the prostate needle biopsy patient set (training), based on (**a**) the four-gene methylation signature (*AOX1*x*GSTP1*x*HAPLN3*x*SLC18A2*), and (**b**) a three-gene model without *GSTP1*. (**c**,**d**) Validation in independent patient sample set analysed on 450 K methylation arrays. Receiver operating characteristic curves for adjacent normal (n = 32; only distant AN samples were included in this analysis for the four patients who also contributed proximal AN samples) vs. non-malignant prostate tissue samples (n = 9), based on (**c**) the four-gene signature (*AOX1*x*GSTP1*x*HAPLN3*x*SLC18A2*), and (**d**) a three-gene model without *GSTP1*. For each gene, we used data from one probe on the 450 K array (*SLC18A2*: cg00498305; *HAPLN3*: cg03628719; *GSTP1*: cg02659086; *AOX1*: cg22953017).

The combined four-gene model (*AOX1*x*GSTP1*x*HAPLN3*x*SLC18A2*) significantly distinguished AN from NM samples (p = 0.0001; χ^2^-test) based on detection of hypermethylation of at least one of the genes in AN tissue. Thus, at 100% fixed specificity, the four-gene methylation signature had 30.8% sensitivity for PC and was able to identify 12 out of 39 PC patients based solely on hypermethylation field effects in AN samples, while not detecting any of the 40 non-cancer patients with exclusively NM biopsies. Notably, the diagnostic accuracy of the four-gene model (AUC = 0.65; Fig. 5A) was superior to PSA (AUC = 0.47; Suppl. Fig. S2) in this patient set (p = 0.01). Importantly, exclusion of *GSTP1* from the model gave highly similar results (AUC = 0.64; Fig. 5B and Suppl. Table S1), indicating that the discriminative power of the four-gene model was not simply driven by *GSTP1* for which hypermethylation cancer field effects have previously been demonstrated in PC^25^,^26^,^28^. Furthermore, with an AUC of 0.64 the three gene model (*AOX1xHAPLN3xSLC18A2*) significantly outperformed (p = 0.006; χ^2^-test) the diagnostic accuracy of *GSTP1* as a single marker (AUC 0.54). There were no significant differences in serum PSA levels between patients with high vs. low methylation in AN tissue for any of the multi-gene models (p = 0.63 (three-gene model) and p = 0.72 (four-gene model); Spearman’s rank test) in this patient set.

In summary, our results support the existence of hypermethylation based field effects in PC and suggest a novel four-gene (*AOX1*x**GSTP1**x*HAPLN3*x*SLC18A2*) epigenetic cancer field effect signature for detection of (occult) PC.

### Validation of epigenetic cancer field effects by microarray analysis of surgical specimens

**Figure 6:**
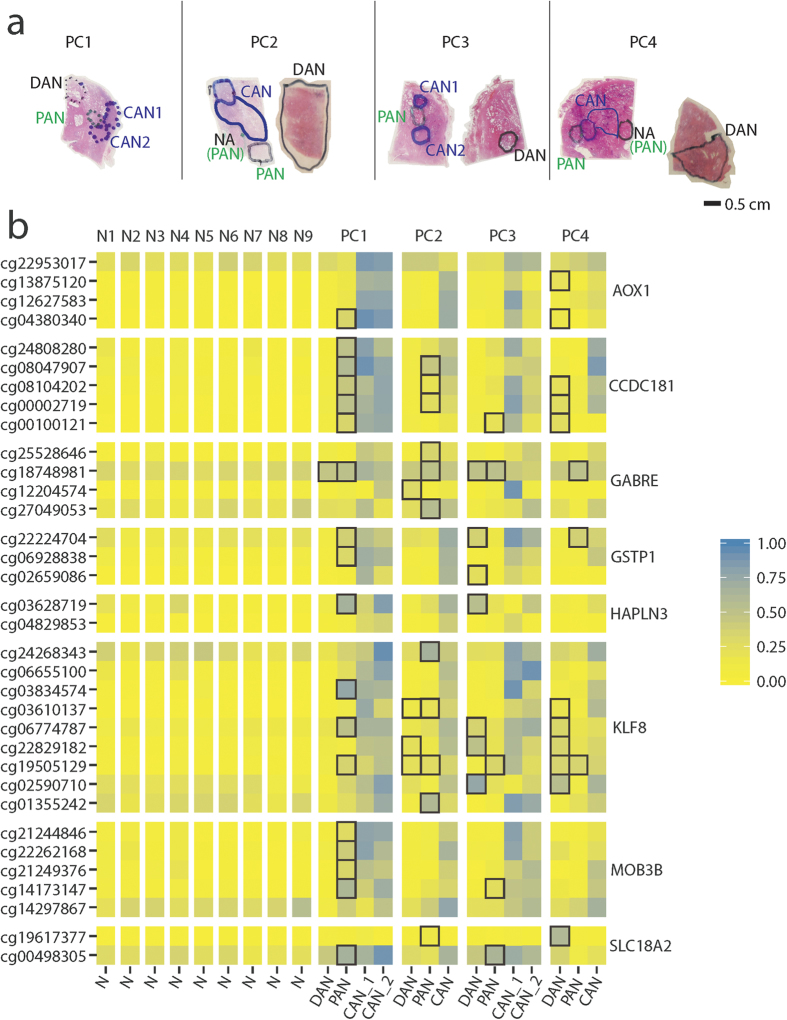
Field effects in the 450 K laser microdissected (LMD) subset. (**a**) Physical location of laser microdissected tissue samples from surgical specimens from four PC patients (PC1-PC4). PAN, proximal adjacent normal; DAN, distant adjacent normal; CAN, cancer foci; NA, not available for this experiment or excluded due to low DNA yield. (**b**) Heatmap of methylation levels in CAN/PAN/DAN samples from four PC patients (PC1-PC4) as compared to normal prostate tissue samples (N1-N9) also analysed on 450 K arrays. Results are shown for probes associated with the CpG islands also analysed by qMSP (see Suppl. Table S5 for further details). Black boxes highlight hypermethylation based field effects, as defined by a beta-value in a DAN/PAN sample that is at least 0.1 higher than the maximum beta-value detected for that particular probe in the normal (N) samples.

To further investigate the existence of epigenetic cancer field effects, we used Illumina 450 K methylation microarray data from an independent prostate tissue sample set of 51 PC patients and 9 controls without prostate cancer (bladder cancer patients) (Suppl. Table S2). At this stage, we analysed whole surgical specimens (prostatectomies) for which a complete histopathological evaluation had been performed, allowing us to map epigenetic cancer field effects in more detail, including from PC patients with verified multifocal disease. Thus, the total sample set used for 450 K analysis included 59 malignant (PC) and 36 adjacent normal (AN) prostate tissue samples from 51 PC patients (22 patient with multiple AN and/or PC samples; 19 patients with one PC sample, and 10 patients with one AN sample), as well as 9 normal (N) prostate tissue samples (Suppl. Table S2). While the majority of samples were macrodissected, four of the PC patients were analysed in more depth after laser microdissection of 1–2 PC foci (cancer samples, CAN), one proximal adjacent normal (PAN) sample (<1 mm from PC), and one distant adjacent normal (DAN) sample (located > 3 mm from PC) (Suppl. Table S3 and Fig. 6a).

For all genes, we focused specifically on DNA methylation levels within the promoter region, as also interrogated by qMSP (*GAS6* was excluded as it had no probes on the array). Significant PC-specific hypermethylation was detected for all genes also in this patient sample set, and mean methylation levels were similar in AN and N samples (Suppl. Fig. S3 and Suppl. Table S4), corroborating our findings in the needle biopsy sample set (Figs. 1 and 4). Next, for each probe, a cut-off for calling hypermethylation field effects in AN samples was defined as a β-value at least 0.1 higher than the maximum β-value for that particular probe in normal (N) samples. Furthermore, to avoid bias, only one AN sample was included in this analysis for each patient (i.e. PAN samples were excluded for the 4 patients also represented by a DAN sample. PAN samples were excluded rather than DAN samples due to the theoretically higher risk of contamination with neighbouring cancer cells in these samples). Using these criteria, we identified a total of 34 probes (CpG sites) for which methylation based field effects were detectable in AN tissue in at least one out of 32 patients analysed (Table 2). For each of the eight genes investigated, epigenetic cancer field effects were detected in a small subset (<20%) of the patients (Table 2), consistent with our finding of heterogeneous and sporadic field effects in diagnostic needle specimens (Fig. 4). Importanty, when reversing the analysis, no probes passed the cut-off, i.e. no probe had a β-value in any of the normal samples that was at least 0.1 higher than the maximum β-value in AN samples (Table 2). This result supports the validity of our findings and indicates that the increased methylation levels observed specifically in AN samples represent cancer-specific field effects rather than random variations in methylation levels between samples.

In the four patients (PC1-PC4) for whom we analysed both a proximal and a distant AN sample, we also observed highly heterogeneous patterns of epigenetic field effects. Thus, in one PC patient (PC1), hypermethylation field effects were almost exclusively detected in the PAN sample, raising the possibility of contamination by hypermethylated cancer cells from the neighboring PC foci (Fig. 6a,b, Suppl. Table S5). In contrast, however, more complex patterns of epigenetic field effects were detected in both DAN and PAN samples in the other three patients (PC2-4), suggesting the existence of a more generalised epigenetic field effect in these cases (Fig. 6a,b, Suppl. Table S5).

Finally, to validate our novel four- and three-gene epigenetic field effect signatures (Fig. 5a,b), we used 450 K methylation array data for the probe located in closest proximity to the qMSP assay used for each gene (for probe IDs, see legends to Fig. 5). The methylation signatures were analysed by the same approach as used for the biopsy (training) set. Thus, for each gene, methylation levels were dichotomised at a cut-off that ensured 100% specificity for AN vs. N samples, and each sample was then scored as hypermethylated, if at least one of the genes in the signature had a methylation level above this cut-off. The four-gene model (*AOX1xGSTP1xHAPLN3xSLC18A2*) had an AUC of 0.70 (sensitivity = 40.6% at 100% fixed specificity) and the three-gene model (*AOX1xHAPLN3xSLC18A2*) had a highly similar AUC of 0.69 (sensitivity = 37.5% at 100% fixed specificity) in the validation set (Fig. 5c,d). Notably, this result also confirmed that the diagnostic performance of our novel four-gene model is not simply driven by the inclusion of *GSTP1*. In comparison, *GSTP1* as a single marker had an AUC of 0.55 in this sample set. In conclusion, we have trained and validated a novel three-gene and a novel four-gene methylation based cancer field effect signature highly specific to PC.

## Discussion

In this study, we show that prostate cancer-specific hypermethylation of the eight genes *AOX1, CCDC181 (C1orf114*), *GABRE, GAS6, HAPLN3, KLF8, MOB3B*, and *SLC18A2* can be detected by qMSP with very high sensitivity and specificity in scarce prostate needle biopsy samples taken at the time of diagnosis. This result provides proof of principle and could pave the way for development of methylation-based molecular diagnostic tests for PC in this clinically relevant context. Furthermore, we report the existence of heterogeneous and sporadic hypermethylation-based cancer field effects for all eight candidate genes (as well as for *GSTP1*) in adjacent non-malignant tissue samples from patients with PC in other biopsies and/or in adjacent non-malignant tissue samples from surgical prostatectomy specimens. Although field effects were detected in only a small proportion of the patients with PC, suggesting limited diagnostic potential of single genes, we trained a novel four-gene (*AOX1*x*GSTP1*x*HAPLN3*x*SLC18A2*) and a novel three-gene (*AOX1*x*HAPLN3*x*SLC18A2*) epigenetic cancer field effect signature that showed 30.8% and 28.2% sensitivity, respectively, at 100% fixed specificity, determined by qMSP analyses of non-malignant prostate biopsy samples from patients with vs. without PC in other biopsies. The four- and three-gene signatures were subsequently validated using 450 K data based on surgical prostatectomy specimens from an independent set of adjacent non-malignant vs. normal prostate tissue samples, resulting in 40.6% and 37.5% sensitivity, respectively, at 100% fixed specificity. Our results warrant further studies of these novel epigenetic cancer field effect signatures to assess their potential future clinical value for PC detection. Notably, while epigenetic field effects have been reported for *GSTP1* in previous PC studies^24^,^25^,^26^,^28^,^29^, this is the first report of cancer field effects for *AOX1, HAPLN3*, and *SLC18A2* in relation to PC.

Based on analyses of RP specimens, we and others have previously shown that *AOX1, CCDC181, GABRE, GAS6, HAPLN3, KLF8, MOB3B*, and *SLC18A2* as well as *GSTP1*, are common targets (AUC > 0.90) of aberrant promoter hypermethylation in PC tissue samples^18^,^20^,^21^,^22^,^23^. In this study, we obtained comparable AUCs, ranging from 0.79 (*GABRE*) to 0.98 (*SLC18A2*), from analyses of malignant vs. non-malignant diagnostic prostate needle biopsies, which not only corroborate the previous reports but also highlight the robustness of our qMSP assays, even for low input DNA samples. Moreover, the significant positive association between methylation levels in PC biopsies and D’Amico risk score for all candidate genes, is also in agreement with previous findings from RP specimens, where high methylation levels were generally associated with at least one adverse clinicopathological factor (high PSA, high Gleason score, positive surgical margins, and/or advanced pT-stage)^20^,^21^,^23^,^43^.

By analysis of surgical specimens from two large RP cohorts, we have previously demonstrated a significant independent prognostic potential for prediction of biochemical recurrence for *GABRE* and *CCDC181* as single methylation markers, as well as for a three-gene methylation signature including *AOX1, CCDC181*, and *HAPLN3*^14^,^20^,^21^. Hence, although this is beyond the scope of our present work, future studies should investigate if the prognostic potential of these top candidate prognostic methylation markers/signature can be transferred to prostate biopsies and thus potentially be used to guide treatment decisions at the time of diagnosis. Importantly, the results of our present study clearly demonstrate that it is possible to perform qMSP-based analysis of several candidate genes in parallel using only leftover biopsy tissue specimens after standard histopathological examination. This may further suggest that it would be relatively easy to incorporate such a test into routine clinical practice in the future.

In addition, future biopsy-based studies could include analysis of *KLF8* and *SLC18A2* for which our previous study in two large patient cohorts showed that a higher methylation level in PC tissue samples from RP specimens was associated with early biochemical recurrence in univariate analyses^20^,^23^. Finally, we note that G*AS6* and *MOB3B* methylation did not show significant prognostic value in our previous study of two large RP cohorts^20^, while the potential prognostic value of *GSTP1* hypermethylation has been evaluated by multiple research groups, however with conflicting results^14^.

The ability to distinguish morphologically normal/non-malignant tissue from cancer tissue in prostate needle biopsies based on DNA methylation analysis probably has limited clinical utility for diagnostic purposes. In contrast, detection of molecular cancer field effects that are not microscopically visible to the pathologist could increase sensitivity for occult PC and ensure early diagnosis of potentially aggressive tumours. PC associated field effects have previously been detected at various molecular levels, including RNA^30^,^31^, protein^33^,^34^ and DNA (mutations)^32^. Here, we focused specifically on epigenetic field effects since aberrant DNA methylation has been found to be an early and highly recurrent event in PC^16^. The existence of methylation based PC field effects has previously been reported based on single gene^25^,^26^,^44^,^45^ as well as genome-wide techniques, including MethylPlex-next-generation sequencing^36^, pyrosequencing^37^, and whole genome bisulphite sequencing^38^. While genomewide approaches may be preferred in the discovery phase, subsequent development of gene-specific qMSP assays (as used in the present study) will allow easier translation into future clinical use, due to their relative simplicity, low cost, and compatibility with standard real-time PCR equipment available in most if not all molecular diagnostic laboratories.

Based on qMSP analysis of malignant and non-malignant prostate needle biopsy specimens, we developed and validated a novel four-gene (*AOX1*x*GSTP1*x*HAPLN3*x*SLC18A2*) epigenetic field effect signature for PC that showed more than 30% sensitivity for PC at 100% specificity. More specifically, the signature was able to identify 12 out of 39 patients with PC based solely on detection of epigenetic field effects in morphologically non-malignant prostate biopsies. Seven of these 12 patients presented with PSA < 10 ng/ml, suggesting that our novel four-gene signature could potentially assist in the detection of PC also in patients with PSA in this lower range. However, further studies are needed to investigate this and to assess whether our epigenetic field effect signature can be used to guide repeat biopsy decisions. The superior performance of multi-gene panels over single markers for detection of PC-associated field effects, as found here, is consistent with a previous report by Brikun *et al*. who suggested a minimum of five hypermethylation markers for detection of occult PC in histologically benign biopsy cores^46^. Hence, future studies should also investigate, if inclusion of additional genes (*e.g. APC, RARB2*, and/or *RASSF1*, see below) may improve the diagnostic performance of our novel epigenetic field effect signature.

Prior to the present study, the most extensively studied multi-gene signatures for detection of DNA methylation-based field effects in diagnostic prostate needle biopsies include various combinations of the four genes *APC, RARB2, GSTP1*, and *RASSF1*^24^,^25^,^26^,^27^,^28^,^29^,^47^. Most notably, this has led to a commercially available test (ConfirmMDx for Prostate Cancer; MDx Health) with a reported negative predictive value of 90% in confirming negative biopsies based on qMSP analysis of *APC, GSTP1*, and *RASSF1*^39^. Since repeat biopsies were not available for our study, we cannot estimate a negative predictive value of our four-gene signature for direct comparison. However, despite reports of relatively high sensitivities (68% and 62%, respectively) for the *APC, GSTP1*, and *RASSF1* field effect signature in two large clinical studies (MATLOC and DOCUMENT), it was at the expense of specificity (64% in both studies)^28^,^29^. Furthermore, the DOCUMENT study reported an AUC of 0.63 for *APC, GSTP1*, and *RASSF1* to distinguish non-malignant prostate needle biopsy samples from patients who later had a positive biopsy vs. those who did not^29^, which is highly similar to the AUCs found in the present study for our four-gene and three-gene epigenetic signatures (0.65 and 0.64, respectively).

Whereas *GSTP1* has been linked with cellular protection from the by-products of oxidative stress^48^, little is known about the possible function of *AOX1, HAPLN3*, and *SLC18A2* in PC. AOX1 has been shown to be involved in degradation of the Imidazo[1,2-*a*]pyrimidine moiety of a specific androgen receptor antagonist, suggesting a possible association with drug sensitivity^49^,^50^. There are no previous reports of HAPLN3 function in relation to cancer, but based on sequence similarity with other proteins, it has been suggested that HAPLN3 might stabilise the hyaluronan:chondroitin sulfate proteoglycan complex that is important for *e.g*. extracellular matrix structure^51^. The *SLC18A2* gene encodes a synaptic vesicular amine transporter protein that has been extensively studied in the central nervous system^52^, while its possible function in PC development and/or progression remains to be investigated.

There are some limitations to our study. Our qMSP results from the biopsy set were based on only one medium-sized patient cohort. Nevertheless, we were able to train novel epigenetic field effect signatures that validated successfully in an independent patient set based on 450 K data from radical prostatectomy specimens. Despite our use of different patient sample types (biopsies vs. surgical specimen) and distinct methods for methylation analysis (qMSP vs 450 K arrays), the novel epigenetic field effect signatures performed equally well in both the training and the validation set, suggesting that they are robust. However, future studies including larger numbers of patients are needed to validate our findings and determine transferable threshold values. Ideally, such future studies should include patients referred to initial as well as repeat prostate biopsy due to suspicion of PC.

In conclusion, our results showed that scarce prostate biopsy tissue sections, leftover after routine histopathological diagnostic procedures, are sufficient for methylation analyses of several candidate genes in parallel. Furthermore, we found frequent and highly prostate cancer-specific hypermethylation of *AOX1, CCDC181, GABRE, GAS6, HAPLN3, KLF8, MOB3B*, and *SLC18A2* in diagnostic needle biopsy samples. We also identified and validated a novel four-gene (*AOX1*x*GSTP1*x*HAPLN3*x*SLC18A2*) epigenetic field effect signature with over 30% sensitivity for PC at 100% fixed specificity. Future studies should investigate if this novel epigenetic field effect signature can be used to increase sensitivity for (occult) PC in a routine clinical setting and/or be used to guide the need for repeat biopsy. If successful, implementation of such a test could help to limit the number of unnecessary repeat biopsies. Finally, to pave the way for non/minimally-invasive diagnostic tests, future studies are also needed to investigate if hypermethylation of *AOX1, CCDC181, GABRE, GAS6, HAPLN3, KLF8, MOB3B*, and/or *SLC18A2* can be detected also in urine and/or blood (plasma/serum) samples from PC patients, as has been reported for *GSTP1*^53^.

## Methods

### Clinical Samples

Two patient sample sets were used in this study for qMSP and 450 K analysis, respectively.

The qMSP set (training): Formalin-fixed and paraffin embedded (FFPE) diagnostic needle biopsies from 176 patients undergoing ultrasound-guided prostate biopsy due to suspicion of PC, were obtained from Department of Pathology, Aarhus University Hospital after routine histopathological examination. As part of standard diagnostic procedures, consecutive 3-μm sections of individual biopsy cores were mounted on glass slides (3–4 sections on each slide from the same biopsy). The total number of cores per patient ranged from 4–24 (with 10 cores being most common) equally representing the left and the right side of the prostate. Leftover sections were stored at −80 °C until used in the present study. For DNA extraction, due to scarce amounts of tissue, biopsy sections were systematically pooled for each patient based on an anatomical left/right separation and a histopathological malignant/non-malignant separation (Suppl. Fig. S1). This led to three sample types: malignant (PC) tissue samples from cancer-positive biopsies, adjacent non-malignant (AN) biopsy samples from patients with PC in at least one other biopsy, and non-malignant (NM) biopsy samples from patients with exclusively cancer-negative biopsies. After exclusion of 31 patients with PIN and/or inflammation in at least one biopsy, a total of 402 samples (114 NM, 109 AN, and 179 malignant samples) from 145 patients were selected for this study. Another 202 samples were excluded from further analysis, due to either low DNA yield, insufficient DNA quality resulting in failed QC of reference genes and/or detection of PC at repeat biopsy within 18 months. The final set consisted of 200 biopsy tissue samples (75 NM, 59 AN, and 66 malignant samples) from 107 patients (Suppl. Fig. S1). Clinicopathological information is provided in Table 1.

The 450 K set (validation): Prostate tissue samples from radical prostatectomy specimens from 51 PC patients who underwent radical prostatectomy for histologically verified PC and from 9 control patients who underwent radical cystoprostatectomy due to bladder cancer (histopathologically confirmed to not have PC). Patient samples were collected at Department of Urology, Aarhus University Hospital, Denmark (2002–2011) and at the Norris Comprehensive Cancer Centre, USA (2010–2011) (see Suppl. Table S2 for clinicopathological information). For 31 PC patients and for 9 bladder cancer patients (controls), we had fresh-frozen TissueTek embedded samples, and for the remaining 20 PC patients we had FFPE tissue samples. In all cases, hematoxylin and eosin (H&E) stained tissue sections were evaluated by an expert histopathologist, who marked areas of interest (AN, adjacent normal; CAN, cancer; N, normal). For DNA extraction we used multiple 5–20 μm unstained sections. Tissue areas of interest were macrodissected, except for 4 PC patients (Suppl. Table S3), who were examined in-depth after lasermicrodissection (LMD) of as many morphologically different, geographically separated PC foci and/or AN samples as possible using whole FFPE prostatectomy specimens. For these 4 PC patients, non-malignant tissue samples were further classified as either distant AN (DAN) or proximal AN (PAN) based on physical distance to the nearest PC focus (>3 mm vs. <1 mm). A total of 14 samples (4 DAN, 4 PAN, and 6 CAN) were included from these 4 patients (Fig. 6a). The total sample set used for 450 K analysis included 59 malignant and 36 adjacent normal prostate tissue samples from 51 PC patients as well as 9 normal (N) prostate tissue samples (Suppl. Table S2).

The study was approved by the Central Denmark Region Committees on Biomedical Research Ethics and The Danish Data Protection Agency. Informed written consent was obtained from all patients. The study was carried out in accordance with the approved guidelines.

### DNA Purification from Tissue

Biopsy tissue sections were adjusted to room temperature for 20 min, deparaffinised in xylene (20 min), and rehydrated in EtOH (99% EtOH for 20 min, 96% EtOH for 10 min, and 70% EtOH for 3 min). Tissue sections were scraped into 1.5 ml tubes and genomic DNA extracted using the QIAamp DNA FFPE Tissue Kit (Qiagen) with the following modifications: Deparaffination was performed as described above and incubation in ATL buffer with proteinase K at 56 °C was extended from 1 to 16 hours. Genomic DNA was extracted from macrodissected fresh-frozen and FFPE prostatectomy specimens using the PUREGENE DNA purification kit (Gentra systems) with proteinase K treatment, and the miRNeasy FFPE kit (Qiagen)/RNeasy plus mini kit (Qiagen), respectively, as described previously^21^,^54^. In addition, FFPE prostatectomy specimens from 4 PC patients were used for laser microdissection (LMD). Tissue sections (5 μm) were mounted on polyethylene naphthalate membrane glass, deparaffinised and rehydrated in xylene/EtOH, and stained with a 0.25% w/v solution of cresyl violet (Sigma-Aldrich) in 99.99% EtOH. The Veritas Microdissection Instrument (Arcturus) was used for LMD of individual DAN/PAN/CAN areas from 10–20 serial sections and genomic DNA extracted using the Allprep DNA/RNA FFPE Kit (Qiagen) or the QIAamp DNA FFPE Tissue Kit (Qiagen).

### Quantitative Methylation Specific PCR

The full procedure is described in Supplementary Methods. Briefly, DNA was bisulphite converted, pre-amplified, and used for qMSP analysis. All qMSP reactions were run in triplicates. Chromosomal locations of qMSP assays are illustrated in Suppl. Fig. S4. The AluC4 assay^55^ (Alu-element based normalisation assay for qMSP) is methylation insensitive and was included for normalisation and quality control. To assess the limit of detection for all candidate genes, we used dilution series (10 ng DNA in total) based on decreasing amounts of methylated control DNA (CpGenome Universal Methylated DNA (Millipore)) diluted into unmethylated control DNA (Whole Genome Amplified DNA). Except for *KLF8*, all qMSP assays performed robustly in up to 200× excess unmethylated DNA, corresponding to detection of <20 methylated copies. The qMSP assay for *KLF8* performed robustly in up to 50× excess unmethylated DNA, corresponding to detection of <80 methylated copies.

### 450 K Methylation Analysis

DNA methylation analyses on the Illumina HumanMethylation450 K BeadChip (450 K) were performed at the Keck School of Medicine, University of Southern California, Los Angeles, USA, or through commercial services provided by The Genome Centre, Barts and the London School of Medicine and Dentistry, London, UK and AROS Applied Biotechnology A/S, Aarhus, Denmark, according to the standard protocol provided by Illumina.

### Data Analysis

Output qMSP data from 7900HT Fast Real-Time PCR System was transferred to Stata 12.1 (StataCorp LP), which was used for all subsequent data analysis. The following QC steps were performed: Samples with quantification cycles (C_q_) >35 for AluC4 were excluded from further analysis. Furthermore, for candidate methylation markers, single outliers more than 2 C_q_ different from the other 2 replicates were excluded, except in cases where C_q_-values from 2 of the 3 replicates exceeded 35. Here all three values were kept. C_q_-values >40 were considered undetermined and the quantity was set to 0. The quantity was also set to 0 for patient samples with a C_q_-value higher than the C_q_-value for WGA (whole-genome amplified DNA; used as unmethylated control) in the same qMSP run. For normalisation, the mean quantity for each candidate gene was divided by the mean quantity for the reference AluC4. Finally, for each patient, the sample with the highest methylation level for each tissue sample type (i.e. NM, AN, or malignant, respectively) was selected to represent that patient, resulting in 40 NM, 39 AN, and 48 malignant samples from a total of 107 biopsy patients for the final analyses.

Differences in methylation levels were assessed using Mann Whitney U tests. Furthermore, for each gene, methylation levels were dichotomised (high/low) based on receiver operator characteristics (ROC) curve analyses with specificity set to 100%. To generate methylation-based multi-gene models for epigenetic cancer field effects, all possible combinations (n = 36) of two-gene models were constructed from the dichotomised data. Based on these results, we also build a four-gene and a three-gene model, relying on the same principle. Thus, for all multi-gene models, patients were classified as belonging to the high-methylation group, if they had high methylation for at least one of the genes in the model. The diagnostic potential of all multi-gene models was assessed using χ^2^-tests. P-values were corrected for multiple testing using Bonferroni correction.

Data from 450 K arrays was analysed in R^56^ where both QC and comparison of methylation levels between samples groups were performed, as described by Morris *et al*.^57^. The methylation level for each probe/CpG site was represented by a β-value, ranging from 0 (fully unmethylated) to 1 (fully methylated). For each gene, probes that passed QC and were located in the promoter associated CpG island analysed by qMSP were selected for further analysis (63 probes). For determination of cancer associated hypermethylation, Mann-Whitney U tests were performed for N samples vs. CAN samples and p-values were adjusted for multiple testing using Bonferroni correction. For analysis of methylation-based cancer field effects, probes with a mean β-value above 0.6 in normal prostate tissue samples were excluded, resulting in a total of 58 probes for further analysis. For each probe, a cut-off for calling hypermethylation field effects in AN samples was defined as a β-value at least 0.1 higher than the maximum β-value for that particular probe in normal (N) samples. For validation of the three-gene and four-gene methylation based field effect signatures, we used β-values for the probe located in closest proximity to the qMSP assay for each gene which had a demonstrated field effect in the 450 K sample set (*SLC18A2*: cg00498305; *HAPLN3*: cg03628719; *GSTP1*: cg02659086; *AOX1*: cg22953017). All analyses of multi-gene models in the validation set were performed in Stata 12.1, as described above for the training (qMSP) set.

P-values <0.05 were considered significant.

## Additional Information

**How to cite this article**: Møller, M. *et al*. Heterogeneous patterns of DNA methylation-based field effects in histologically normal prostate tissue from cancer patients. *Sci. Rep.* **7**, 40636; doi: 10.1038/srep40636 (2017).

**Publisher's note:** Springer Nature remains neutral with regard to jurisdictional claims in published maps and institutional affiliations.

## Supplementary Material

Supplementary Information

## Figures and Tables

**Table 1:** Clinicopathological characteristic for patients undergoing prostate biopsy.

Patients		NM	AN¤	Malignant
Number	107§	40	39	48
Age, years	Median (range)	65 (44–79)	64 (50–85)	70 (53–86)
PSA, ng/mL (%)	≤10	22 (55)	23 (59)	18 (38)
	>10 to 20	12 (30)	12 (31)	16 (33)
	>20	5 (12.5)	4 (10)	14 (29)
	Unknown	1 (2.5)	0	0
	Median (range)	8.4 (0.8–46)	9.0 (2.3–78)	13.0 (2.3–856)
Gleason score (%)	6	—	27 (69)	13 (27)
	7	—	11 (28)	16 (33)
	≥8	—	1 (3)	19 (40)
cT (%)*	cT1c or cT2a	—	35 (90)	26 (54)
	cT2c-cT4	—	3 (7.5)	19 (40)
	Unknown	—	1 (2.5)	3 (6)
D’Amico risk (%)#	Low	—	18 (46)	7 (15)
	Intermediate	—	16 (41)	15 (31)
	High	—	5 (13)	26 (54)

^¤^Gleason score, cT stage, and D’Amico risk refers to malignant findings in biopsies from the same patient.

^§^20 patients were represented with both a malignant as well as an adjacent normal (AN) biopsy tissue sample.

^*^Clinical tumour stage (cT) determined by transrectal ultrasound, digital rectal examination, and presence of cancer in prostate needle biopsies.

^#^Low risk (PSA ≤ 10 ng/mL, and Gleason score ≤6, and cT1c/cT2a), intermediate risk (PSA > 10 to 20 ng/mL, and/or Gleason score 7, and/or cT2b), high risk (PSA > 20 ng/mL, and/or Gleason score 8–10, and/or cT2c-cT4).

**Table 2:** Epigenetic cancer field effects in patient sample set analysed on 450 K arrays.

Gene	Probe ID	N β Range	AN β Range	Field effects (AN > N) (%, n = 32)	N > AN (%, n = 9)
*AOX1*	cg22953017	0.24; 0.38	0.17; 0.51	**1 (3.12)**	0 (0)
*AOX1*	cg13875120	0.01; 0.07	0; 0.21	**2 (6.25)**	0 (0)
*AOX1*	cg12627583	0.03; 0.1	0.03; 0.2	**1 (3.12)**	0 (0)
*AOX1*	cg04380340	0.01; 0.04	0.01; 0.16	**1 (3.12)**	0 (0)
*CCDC181*	cg24808280	0.03; 0.17	0.03; 0.33	**1 (3.12)**	0 (0)
*CCDC181*	cg08047907	0.02; 0.12	0.02; 0.25	**2 (6.25)**	0 (0)
*CCDC181*	cg08104202	0.02; 0.09	0.03; 0.24	**3 (9.38)**	0 (0)
*CCDC181*	cg00002719	0; 0.08	0; 0.27	**1 (3.12)**	0 (0)
*CCDC181*	cg00100121	0; 0.05	0; 0.26	**1 (3.12)**	0 (0)
*GABRE*	cg25528646	0.02; 0.13	0.02; 0.3	**1 (3.12)**	0 (0)
*GABRE*	cg18748981	0.28; 0.37	0.19; 0.52	***4 (12.5)***	0 (0)
*GABRE*	cg12204574	0.02; 0.1	0.01; 0.3	**1 (3.12)**	0 (0)
*GABRE*	cg27049053	0.14; 0.4	0.11; 0.56	**1 (3.12)**	0 (0)
*GSTP1*	cg22224704	0.13; 0.27	0.08; 0.37	**1 (3.12)**	0 (0)
*GSTP1*	cg06928838	0.04; 0.14	0.03; 0.3	**1 (3.12)**	0 (0)
*GSTP1*	cg02659086	0; 0.05	0; 0.24	**1 (3.12)**	0 (0)
*HAPLN3*	cg04829853	0; 0.03	0.01; 0.18	**1 (3.12)**	0 (0)
*HAPLN3*	cg03628719	0.03; 0.31	0.05; 0.54	**1 (3.12)**	0 (0)
*KLF8*	cg24268343	0.18; 0.45	0.1; 0.59	**1 (3.12)**	0 (0)
*KLF8*	cg06655100	0.03; 0.23	0.03; 0.48	**3 (9.38)**	0 (0)
*KLF8*	cg03834574	0.02; 0.12	0.02; 0.45	**2 (6.25)**	0 (0)
*KLF8*	cg03610137	0.01; 0.04	0.02; 0.34	***5 (15.62)***	0 (0)
*KLF8*	cg06774787	0.12; 0.17	0.11; 0.43	**3 (9.38)**	0 (0)
*KLF8*	cg22829182	0.04; 0.13	0.04; 0.51	***6 (18.75)***	0 (0)
*KLF8*	cg19505129	0.02; 0.1	0.04; 0.45	***6 (18.75)***	0 (0)
*KLF8*	cg02590710	0.13; 0.29	0.07; 0.75	***6 (18.75)***	0 (0)
*KLF8*	cg01355242	0.17; 0.36	0.03; 0.53	**1 (3.12)**	0 (0)
*MOB3B*	cg21244846	0.02; 0.14	0.01; 0.27	**2 (6.25)**	0 (0)
*MOB3B*	cg22262168	0.03; 0.16	0.02; 0.35	**1 (3.12)**	0 (0)
*MOB3B*	cg21249376	0.01; 0.19	0.01; 0.38	**1 (3.12)**	0 (0)
*MOB3B*	cg14173147	0.03; 0.11	0.04; 0.25	**1 (3.12)**	0 (0)
*MOB3B*	cg14297867	0.21; 0.55	0.09; 0.68	**1 (3.12)**	0 (0)
*SLC18A2*	cg00498305	0.17; 0.36	0.1; 0.6	**2 (6.25)**	0 (0)
*SLC18A2*	cg19617377	0.01; 0.04	0.01; 0.6	**1 (3.12)**	0 (0)

N: normal prostate tissue samples from cystoprostatectomy patients from the 450 K set. AN: adjacent normal prostate tissue samples (PAN samples excluded for the four patients also represented by a DAN sample). Field effects AN > N: the total number of patients with a field effect detected for a given probe based on the criteria:

Any AN β-value at least 0.1 higher than the maximum β-value for that particular probe in N. N > AN: The number of N samples with hypermethylation compared to AN samples (any N sample with a β-value at least 0.1 higher than the maximum β-value for that particular probe in AN samples).

Field effects detected in ≤10% of the patients are marked in bold.

Field effects detected in 10-25% of the patients are marked in bold and italics.
