# Supplementary material for: Heterogeneous patterns of DNA methylation-based field effects in histologically normal prostate tissue from cancer patients
Source: Sci Rep. 2017 Jan 13;7:40636. doi: 10.1038/srep40636 (PMC5233981; doi:10.1038/srep40636)
Supplement: Supplementary Information [file srep40636-s1.pdf]

Supplementary Information

Heterogeneous patterns of DNA methylation-based field effects in histologically normal prostate tissue from cancer patients

Mia Møller[1], Siri Hundtofte Strand[1], Kamilla Mundbjerg[2], Gangning Liang[2], Inderbir Gill[2], Christa Haldrup[1], Michael Borre[3], Søren Høyer[4], Torben Falck Ørntoft[1], Karina Dalsgaard Sørensen[1]*.

[1]Department of Molecular Medicine, Aarhus University Hospital, Aarhus, Denmark
[2]Keck School of Medicine of University of Southern California, Los Angeles, California
[3]Department of Urology, Aarhus University Hospital, Aarhus, Denmark
[4]Department of Pathology, Aarhus University Hospital, Aarhus, Denmark

Supplementary Figures

Supplementary Figure S1
Overview of sample selection for the prostate biopsy patient set.
Samples were stratified based on an anatomical left/right separation and thereafter on histological malignant/non-malignant status in individual biopsy cores (a). Exclusion of samples was performed due to morphological composition of the samples, insufficient amounts of DNA, poor performance in the qMSP assays or detection of cancer in repeat biopsies within 18 months (b).

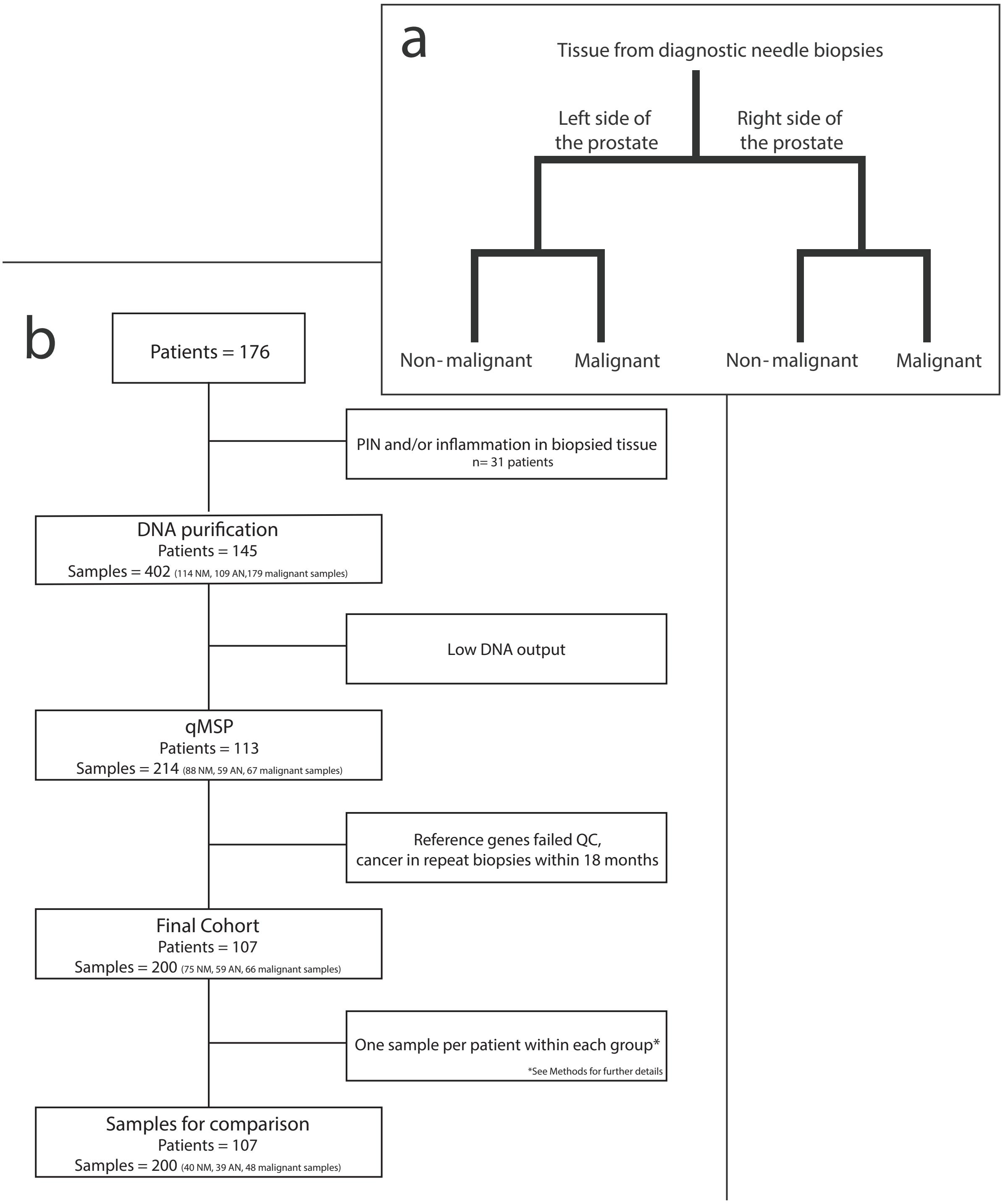

Supplementary Figure S2

Ability of PSA to distinguish adjacent normal (AN) samples and non-malignant (NM) samples in the biopsy patient set. Receiver operating characteristics (ROC) curves for 39 adjacent normal samples and 40 non-malignant samples**.** The included diagonal line corresponds to no difference.

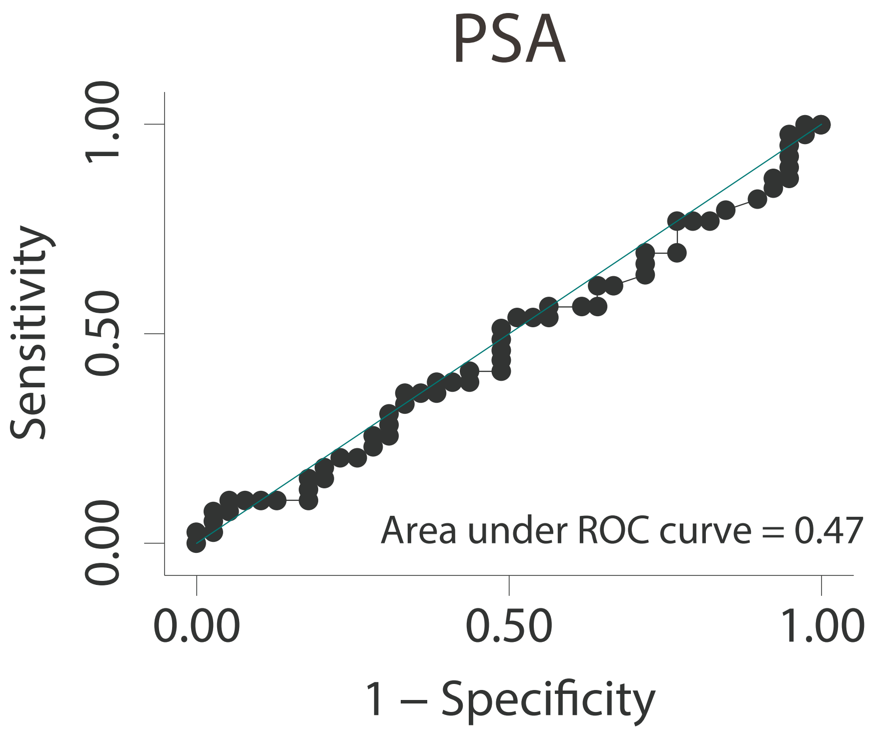

Supplementary Figure S3

Methylation levels in chromosomal regions surrounding the CpG sites analysed by qMSP.

Methylation status in adjacent normal (AN, n = 32, orange line), normal (N, n = 9, blue line) and prostate cancer tissue samples (CAN, n = 59, black line) from the patient sample set analysed on 450K arrays**.** Probe sites are indicated by grey triangles. Black triangles illustrate probes with a significant difference in beta values between N and CAN samples (adjusted p-value < 0.05 in Mann-Whitney U test). Likewise, the red triangle illustrates a probe site with significant difference between AN and N samples. For each gene, the chromosomal location of the qMSP assay is indicated by a bold red line at the top of the plot. For further information about probe IDs and methylation levels, see Supplementary Table S4.

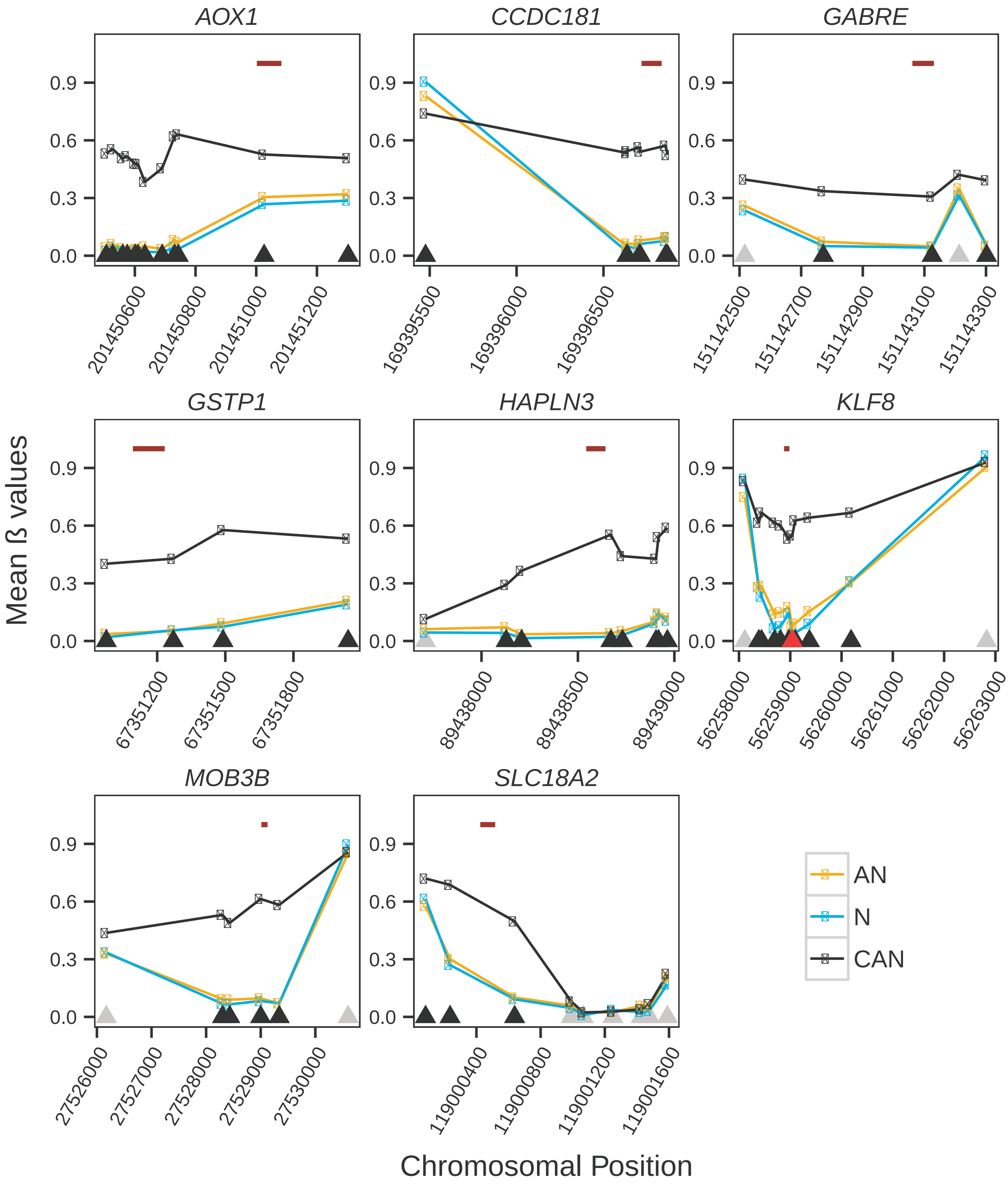

Supplementary Figure S4

Genomic location of qMSP assays for all 9 genes investigated.

Screenshots from USCS genome browser (GRCh37/hg19) with relevant tracks retained. qMSP area (black), genomic structure (blue), CpG island location (green) and 450K probe placement (various colours) are illustrated for the entire gene (a) and zoomed in on the qMSP regions (b).

AOX1

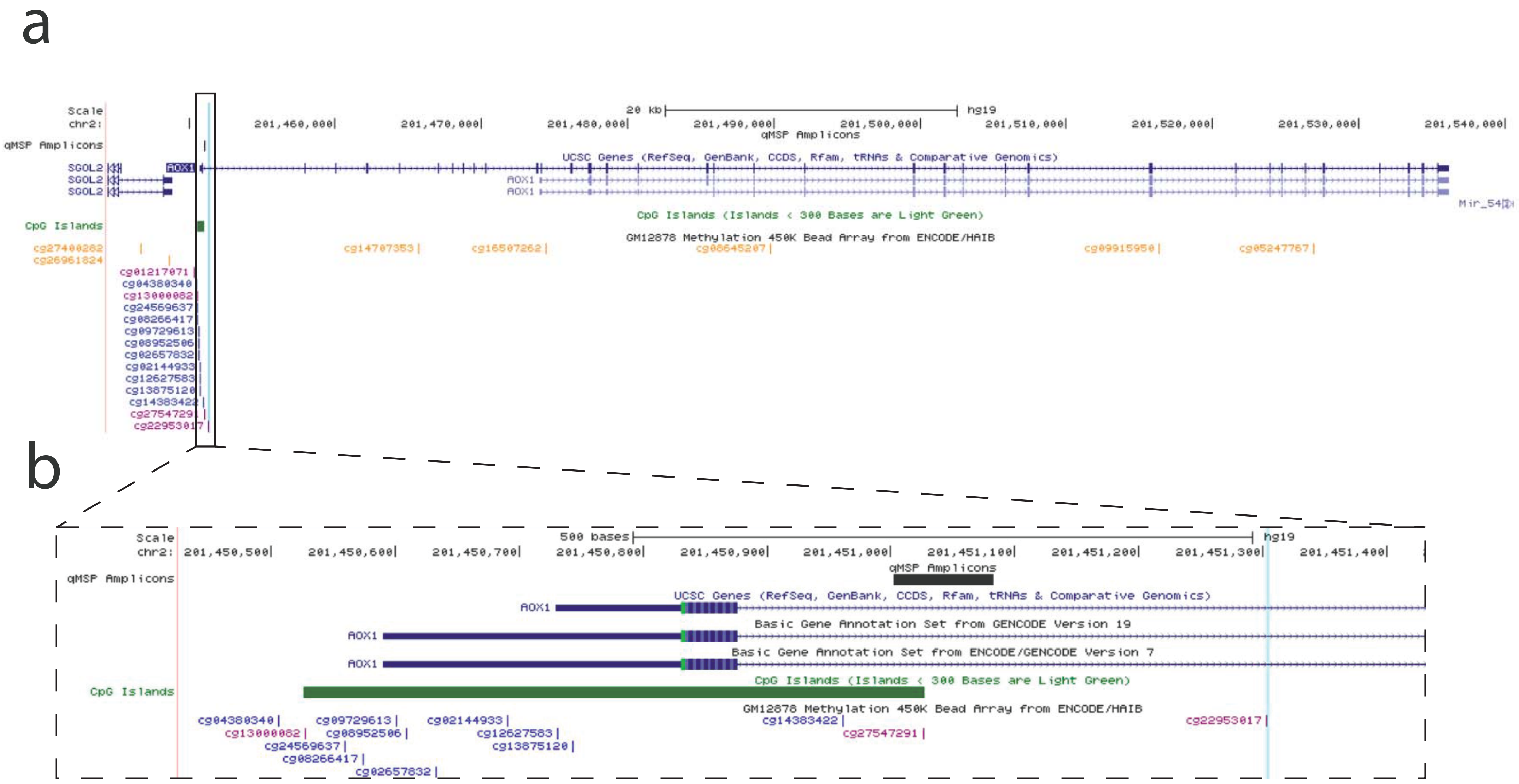
a
Scale
chr2:
20 kb
hg19
201,460,000
201,470,000
201,480,000
201,490,000
201,500,000
201,510,000
201,520,000
201,530,000
201,540,000
qMSP Amplicons
UCSC Genes (RefSeq, GenBank, CCDS, Rfam, tRNAs & Comparative Genomics)
SGOL2
SGOL2
SGOL2
AOX1
AOX1
AOX1
Mir_54
CpG Islands
CpG Islands (Islands < 300 Bases are Light Green)
GM12878 Methylation 450K Bead Array from ENCODE/HAIB
cg27400282
cg26961824
cg14707353
cg16507262
cg08645207
cg09915950
cg05247767
cg01217071
cg04380340
cg13000082
cg24569637
cg08266417
cg09729613
cg08952506
cg02657832
cg02144933
cg12627583
cg13875120
cg14383422
cg27547291
cg22953017
b
Scale
chr2:
500 bases
hg19
201,450,500
201,450,600
201,450,700
201,450,800
201,450,900
201,451,000
201,451,100
201,451,200
201,451,300
201,451,400
qMSP Amplicons
UCSC Genes (RefSeq, GenBank, CCDS, Rfam, tRNAs & Comparative Genomics)
AOX1
Basic Gene Annotation Set from GENCODE Version 19
AOX1
Basic Gene Annotation Set from ENCODE/GENCODE Version 7
AOX1
CpG Islands
CpG Islands (Islands < 300 Bases are Light Green)
GM12878 Methylation 450K Bead Array from ENCODE/HAIB
cg04380340
cg13000082
cg24569637
cg08266417
cg09729613
cg08952506
cg02657832
cg02144933
cg12627583
cg13875120
cg14383422
cg27547291
cg22953017

CCDC181

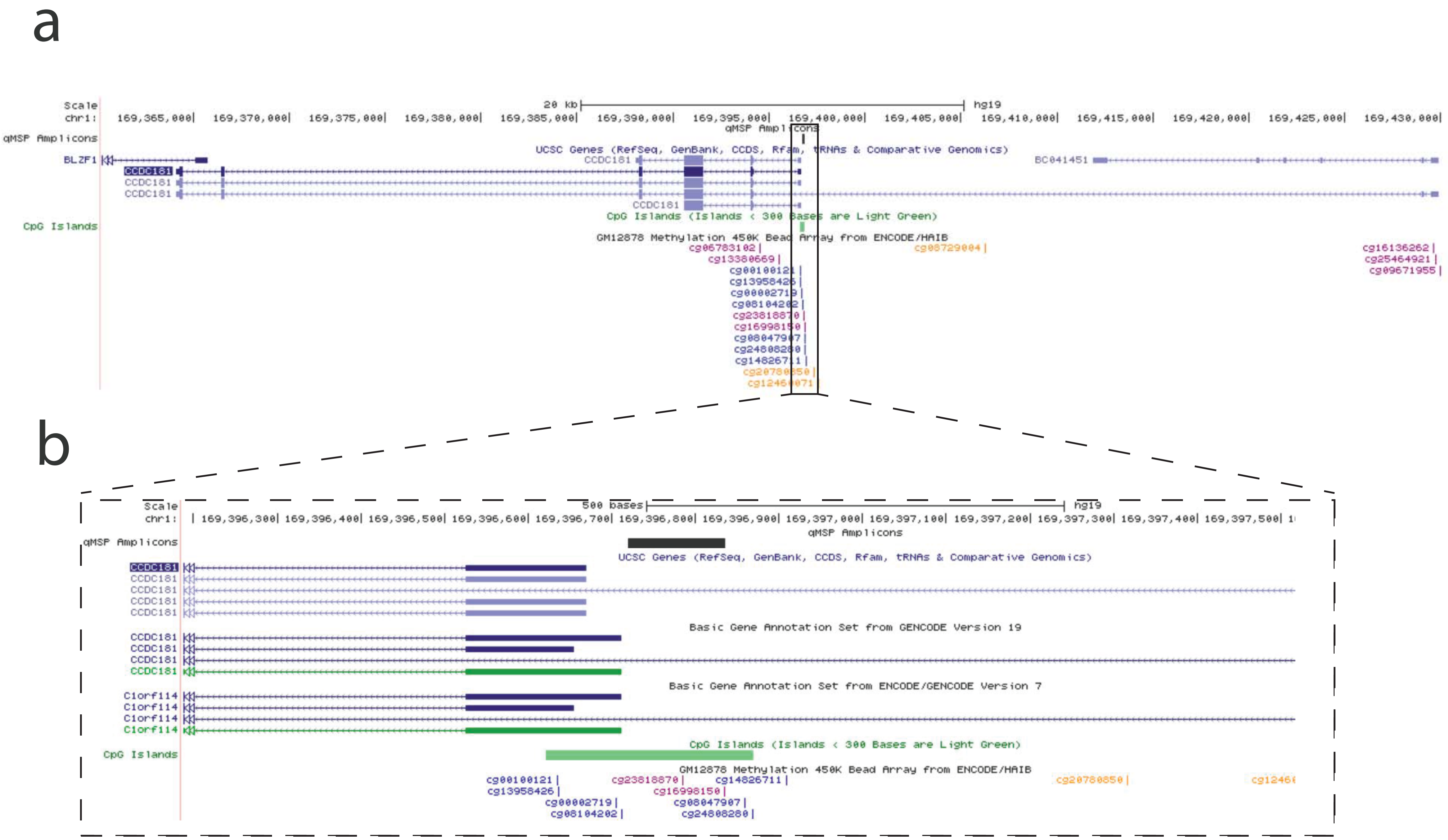

a
Scale
chr1:
20 kb
hg19
169,365,000 169,370,000 169,375,000 169,380,000 169,385,000 169,390,000 169,395,000 169,400,000 169,405,000 169,410,000 169,415,000 169,420,000 169,425,000 169,430,000
qMSP Amplicons
UCSC Genes (RefSeq, GenBank, CCDS, Rfam, tRNAs & Comparative Genomics)
BLZF1
CCDC181
BC041451
CpG Islands
CpG Islands (Islands < 300 Bases are Light Green)
GM12878 Methylation 450K Bead Array from ENCODE/HAIB
cg06783102
cg13380669
cg08729004
cg16136262
cg25464921
cg09671955
b
Scale
chr1:
500 bases
hg19
169,396,300 169,396,400 169,396,500 169,396,600 169,396,700 169,396,800 169,396,900 169,397,000 169,397,100 169,397,200 169,397,300 169,397,400 169,397,500
qMSP Amplicons
UCSC Genes (RefSeq, GenBank, CCDS, Rfam, tRNAs & Comparative Genomics)
CCDC181
Basic Gene Annotation Set from GENCODE Version 19
Basic Gene Annotation Set from ENCODE/GENCODE Version 7
C1orf114
CpG Islands
CpG Islands (Islands < 300 Bases are Light Green)
GM12878 Methylation 450K Bead Array from ENCODE/HAIB
cg00100121
cg13958426
cg00002719
cg08104202
cg23818870
cg16998150
cg14826711
cg08047907
cg24808280
cg20780850

GABRE

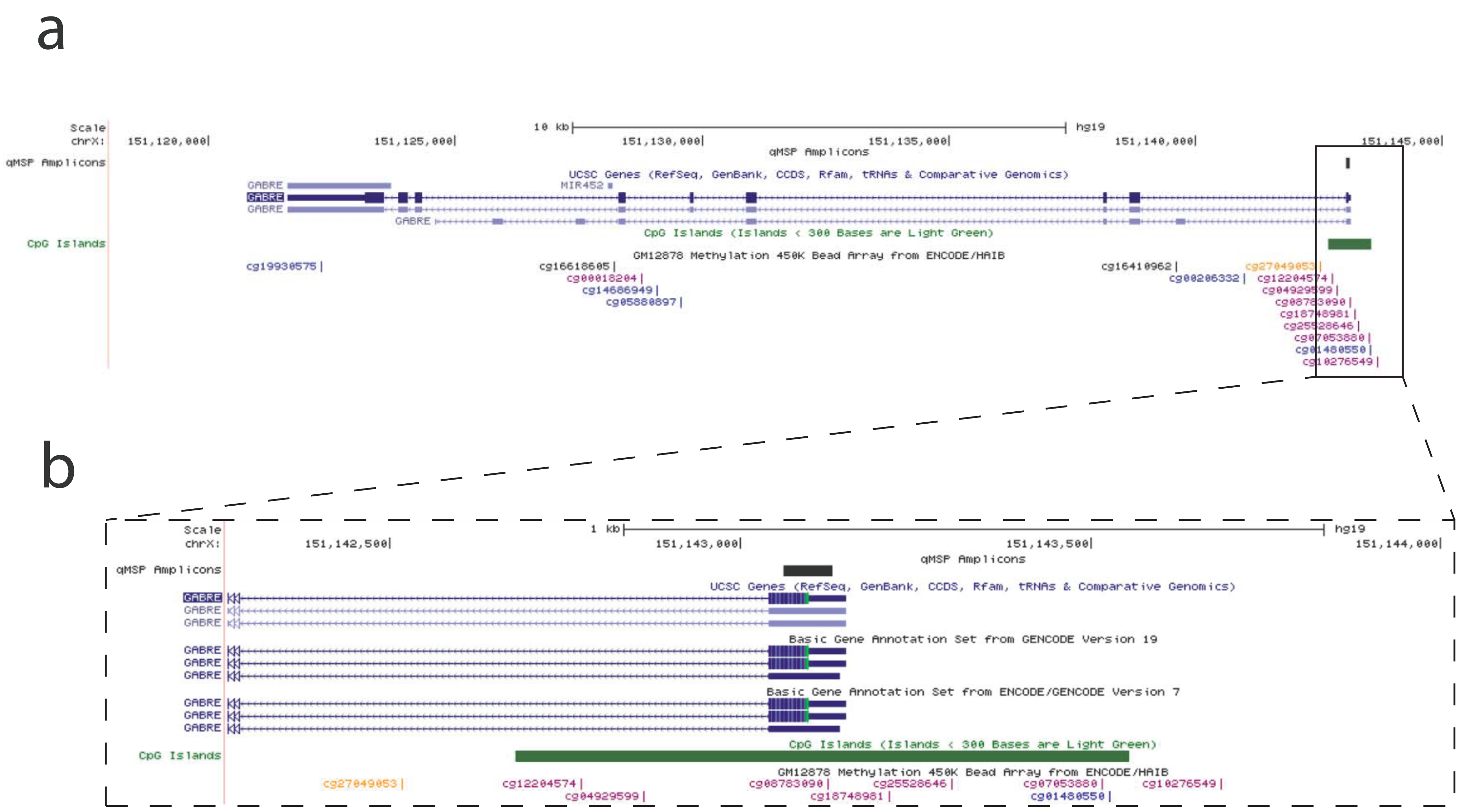
a
Scale
chrX:
10 kb
hg19
151,120,000
151,125,000
151,130,000
151,135,000
151,140,000
151,145,000
qMSP Amplicons
UCSC Genes (RefSeq, GenBank, CCDS, Rfam, tRNAs & Comparative Genomics)
GABRE
MIR452
CpG Islands (Islands < 300 Bases are Light Green)
CpG Islands
GM12878 Methylation 450K Bead Array from ENCODE/HAIB
cg19930575
cg16618605
cg00018204
cg14686949
cg05880897
cg16410962
cg00206332
cg27049053
cg12204574
cg04929599
cg08783090
cg18748981
cg25528646
cg07053880
cg01480550
cg10276549
b
1 kb
151,142,500
151,143,000
151,143,500
151,144,000
Basic Gene Annotation Set from GENCODE Version 19
Basic Gene Annotation Set from ENCODE/GENCODE Version 7

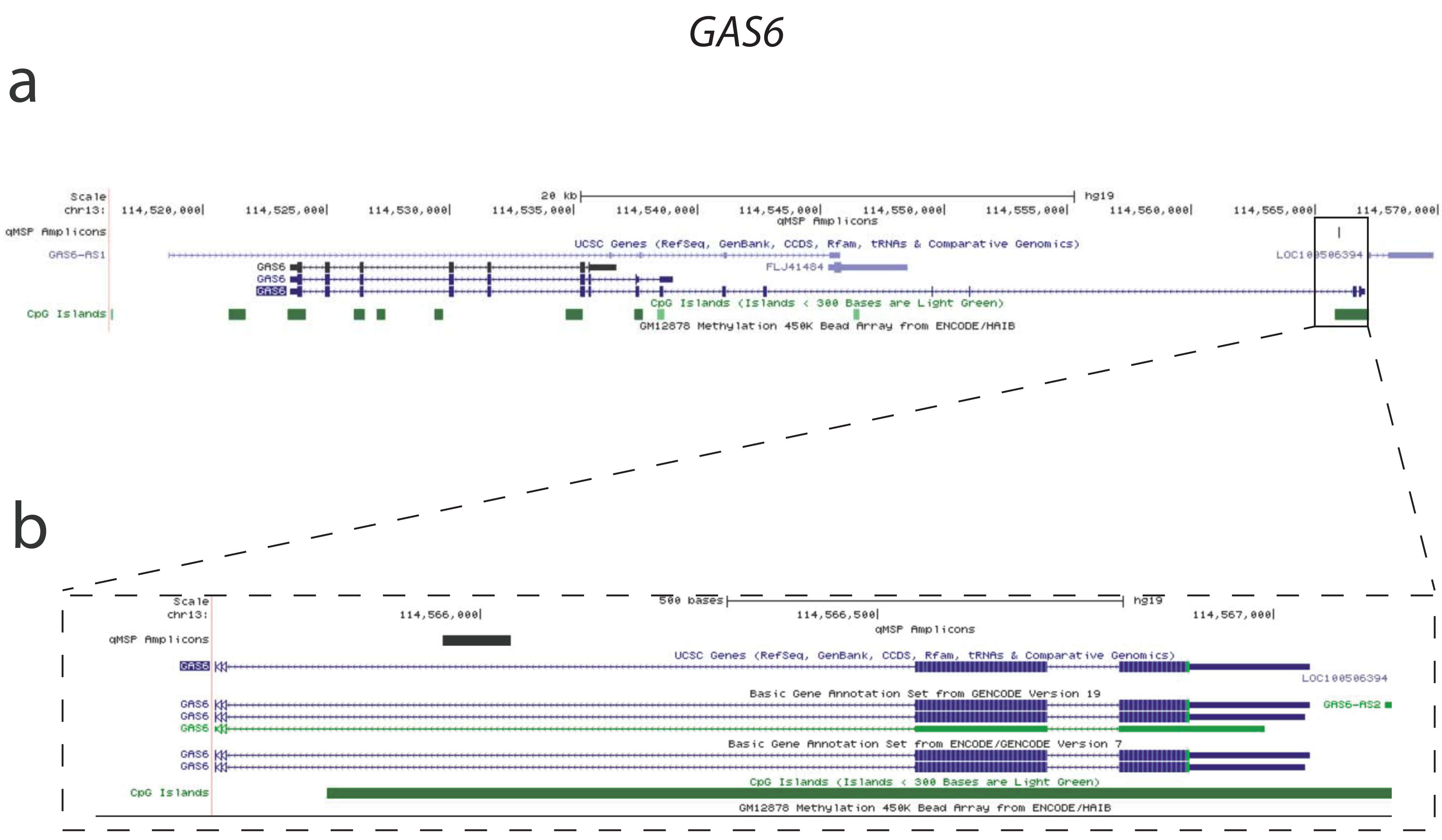
GAS6
a
Scale
chr13:
20 kb
hg19
114,520,000
114,525,000
114,530,000
114,535,000
114,540,000
114,545,000
114,550,000
114,555,000
114,560,000
114,565,000
114,570,000
qMSP Amplicons
UCSC Genes (RefSeq, GenBank, CCDS, Rfam, tRNAs & Comparative Genomics)
GAS6-AS1
GAS6
GAS6
GAS6
FLJ41484
LOC100506394
CpG Islands
CpG Islands (Islands < 300 Bases are Light Green)
GM12878 Methylation 450K Bead Array from ENCODE/HAIB
b
Scale
chr13:
500 bases
hg19
114,566,000
114,566,500
114,567,000
qMSP Amplicons
UCSC Genes (RefSeq, GenBank, CCDS, Rfam, tRNAs & Comparative Genomics)
GAS6
LOC100506394
Basic Gene Annotation Set from GENCODE Version 19
GAS6
GAS6
GAS6
GAS6-AS2
Basic Gene Annotation Set from ENCODE/GENCODE Version 7
GAS6
GAS6
CpG Islands
CpG Islands (Islands < 300 Bases are Light Green)
GM12878 Methylation 450K Bead Array from ENCODE/HAIB

HAPLN3

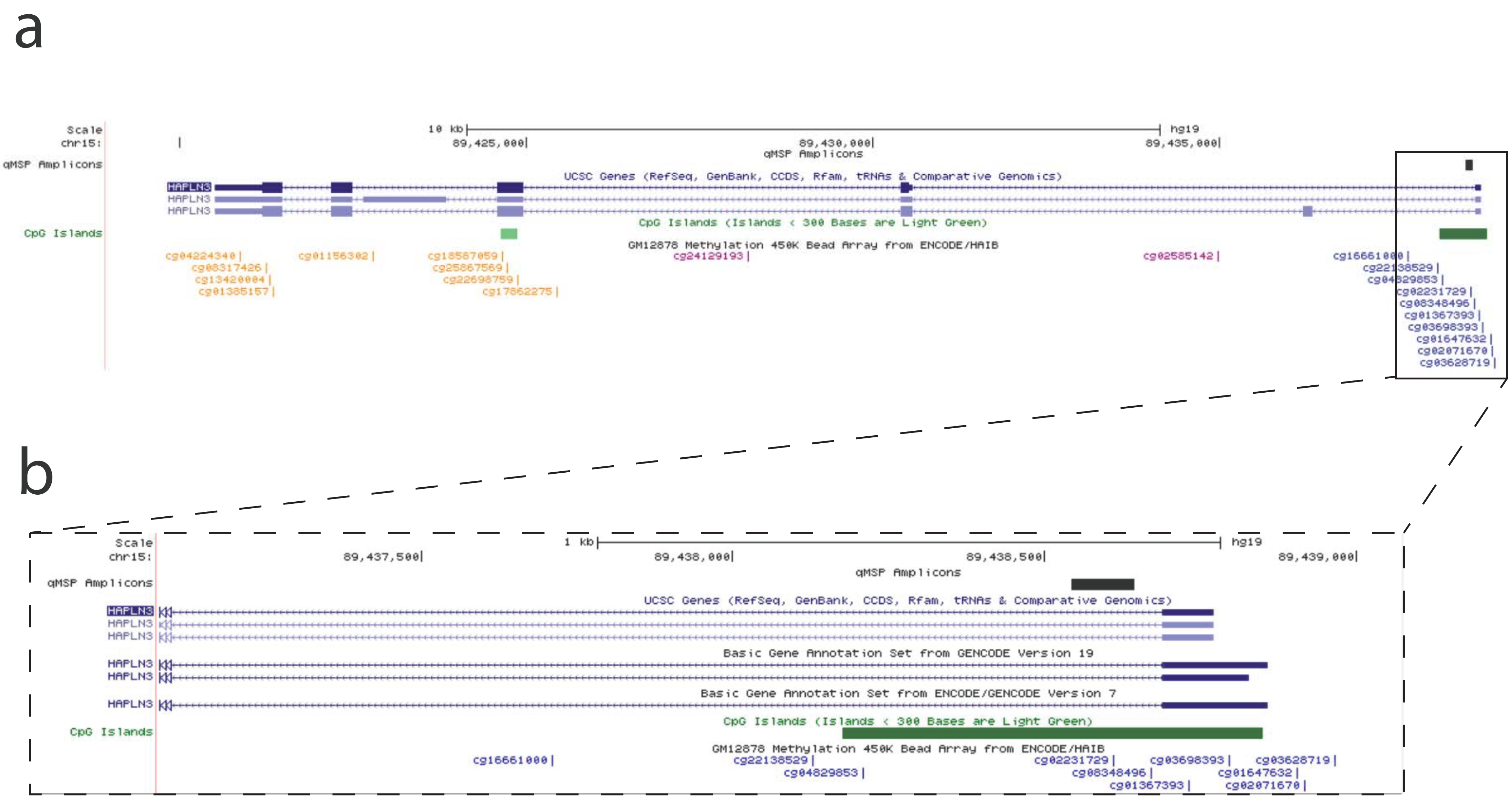
a
Scale
chr15:
qMSP Amplicons
10 kb
hg19
89,425,000
89,430,000
89,435,000
qMSP Amplicons
UCSC Genes (RefSeq, GenBank, CCDS, Rfam, tRNAs & Comparative Genomics)
HAPLN3
HAPLN3
HAPLN3
CpG Islands
CpG Islands (Islands < 300 Bases are Light Green)
GM12878 Methylation 450K Bead Array from ENCODE/HAIB
cg04224340
cg08317426
cg13420004
cg01385157
cg01156302
cg18587059
cg25867569
cg22698759
cg17862275
cg24129193
cg02585142
cg16661000
cg22138529
cg04829853
cg02231729
cg08348496
cg01367393
cg03698393
cg01647632
cg02071670
cg03628719
b
Scale
chr15:
qMSP Amplicons
1 kb
hg19
89,437,500
89,438,000
89,438,500
89,439,000
qMSP Amplicons
UCSC Genes (RefSeq, GenBank, CCDS, Rfam, tRNAs & Comparative Genomics)
HAPLN3
HAPLN3
HAPLN3
Basic Gene Annotation Set from GENCODE Version 19
HAPLN3
HAPLN3
Basic Gene Annotation Set from ENCODE/GENCODE Version 7
HAPLN3
CpG Islands
CpG Islands (Islands < 300 Bases are Light Green)
GM12878 Methylation 450K Bead Array from ENCODE/HAIB
cg16661000
cg22138529
cg04829853
cg02231729
cg08348496
cg01367393
cg03698393
cg01647632
cg02071670
cg03628719

KLF8

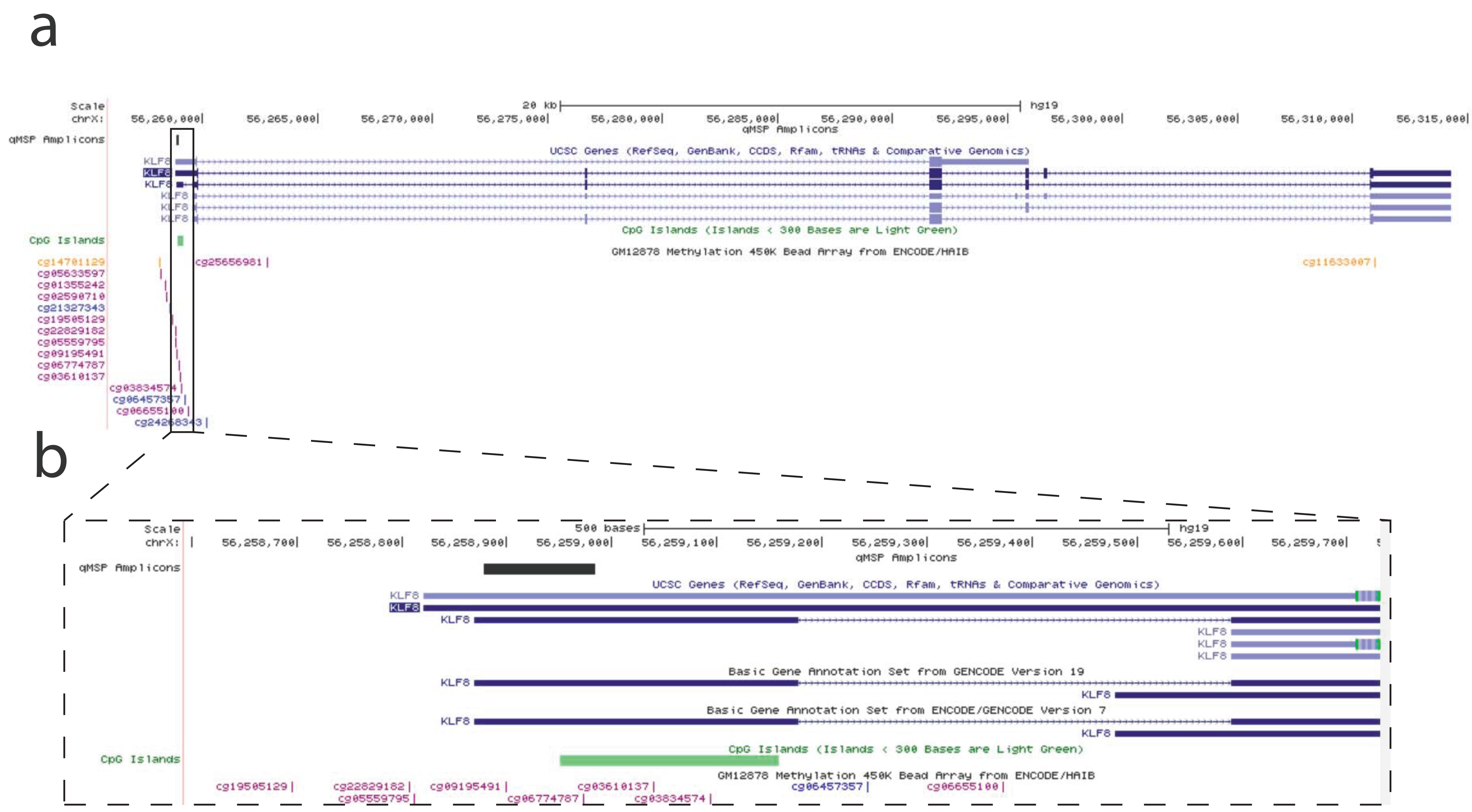

a
Scale
chrX:
20 kb
hg19
56,260,000 56,265,000 56,270,000 56,275,000 56,280,000 56,285,000 56,290,000 56,295,000 56,300,000 56,305,000 56,310,000 56,315,000
qMSP Amplicons
UCSC Genes (RefSeq, GenBank, CCDS, Rfam, tRNAs & Comparative Genomics)
KLF8
CpG Islands
CpG Islands (Islands < 300 Bases are Light Green)
GM12878 Methylation 450K Bead Array from ENCODE/HAIB
cg14701129
cg05633597
cg01355242
cg02590710
cg21327343
cg19505129
cg22829182
cg05559795
cg09195491
cg06774787
cg03610137
cg03834574
cg06457357
cg06655100
cg24268343
cg25656981
cg11633007
b
Scale
chrX:
500 bases
hg19
56,258,700 56,258,800 56,258,900 56,259,000 56,259,100 56,259,200 56,259,300 56,259,400 56,259,500 56,259,600 56,259,700
qMSP Amplicons
UCSC Genes (RefSeq, GenBank, CCDS, Rfam, tRNAs & Comparative Genomics)
KLF8
Basic Gene Annotation Set from GENCODE Version 19
Basic Gene Annotation Set from ENCODE/GENCODE Version 7
CpG Islands
CpG Islands (Islands < 300 Bases are Light Green)
GM12878 Methylation 450K Bead Array from ENCODE/HAIB
cg19505129
cg22829182
cg05559795
cg09195491
cg06774787
cg03610137
cg03834574
cg06457357
cg06655100

MOB3B

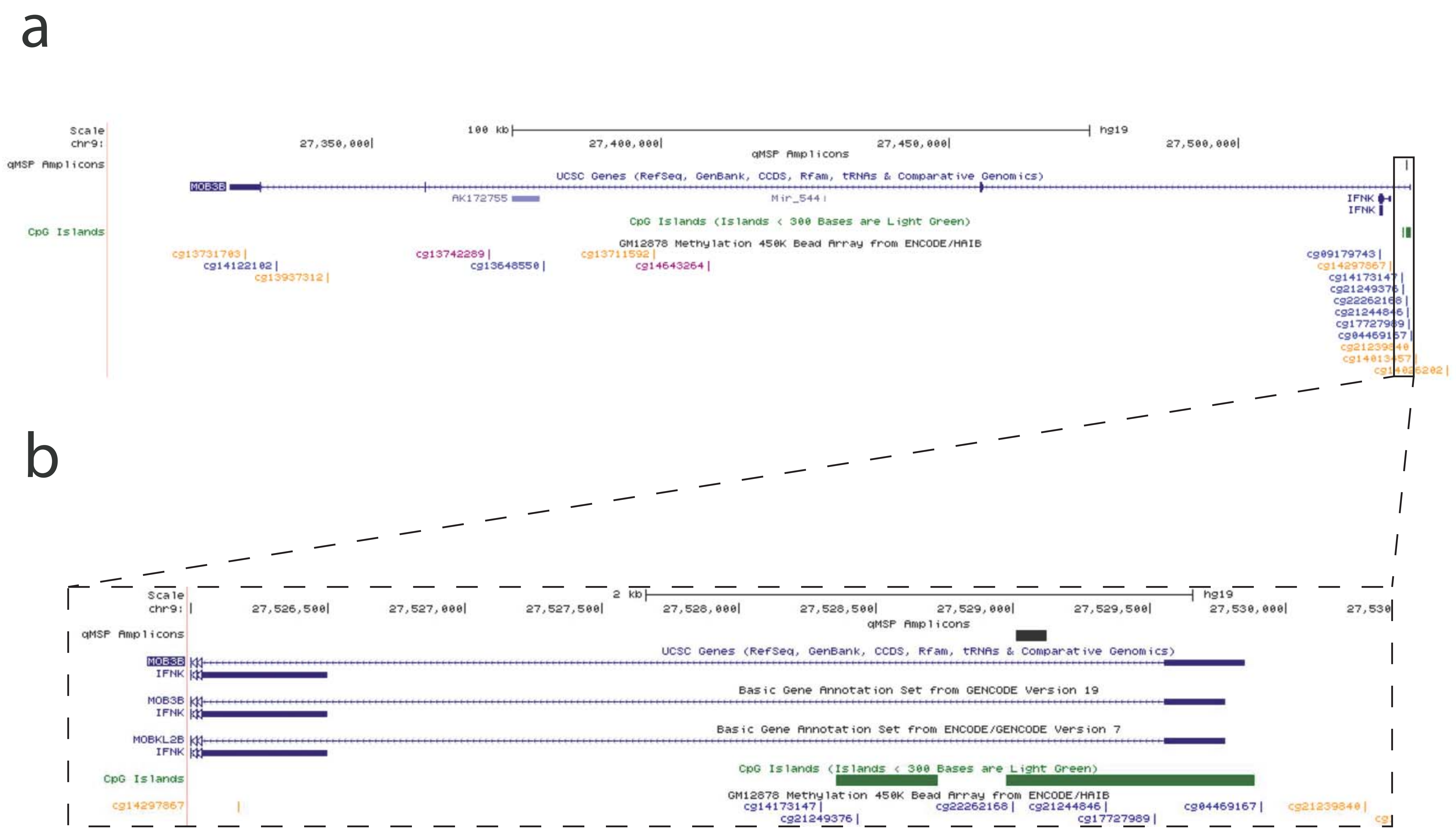
a
b
Scale
chr9:
qMSP Amplicons
100 kb
hg19
27,350,000
27,400,000
27,450,000
27,500,000
UCSC Genes (RefSeq, GenBank, CCDS, Rfam, tRNAs & Comparative Genomics)
MOB3B
AK172755
Mir_544
IFNK
CpG Islands
CpG Islands (Islands < 300 Bases are Light Green)
GM12878 Methylation 450K Bead Array from ENCODE/HAIB
cg13731703
cg14122102
cg13937312
cg13742289
cg13648550
cg13711592
cg14643264
cg09179743
cg14297867
2 kb
27,526,500
27,527,000
27,527,500
27,528,000
27,528,500
27,529,000
27,529,500
27,530,000
Basic Gene Annotation Set from GENCODE Version 19
Basic Gene Annotation Set from ENCODE/GENCODE Version 7
MOBKL2B
cg14173147
cg22262168
cg21244846
cg04469167
cg21239840
cg21249376
cg17727989

SLC18A2

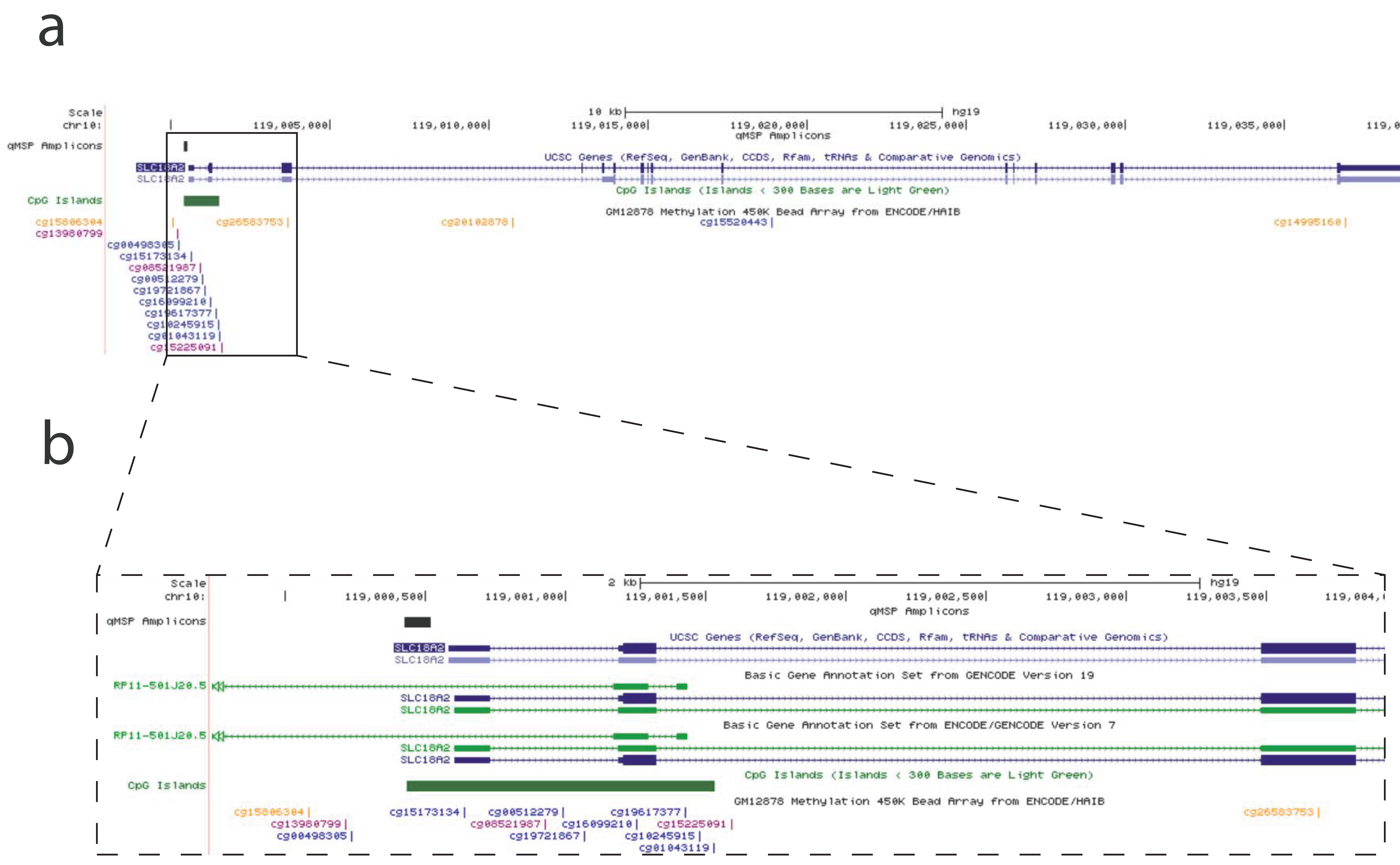

a
Scale
chr10:
10 kb
hg19
119,005,000
119,010,000
119,015,000
119,020,000
119,025,000
119,030,000
119,035,000
119,040,000
qMSP Amplicons
UCSC Genes (RefSeq, GenBank, CCDS, Rfam, tRNAs & Comparative Genomics)
SLC18A2
CpG Islands
CpG Islands (Islands < 300 Bases are Light Green)
GM12878 Methylation 450K Bead Array from ENCODE/HAIB
cg15806304
cg13980799
cg00498305
cg15173134
cg08521987
cg00512279
cg19721867
cg16099210
cg19617377
cg10245915
cg01043119
cg15225091
cg26583753
cg20102878
cg15520443
cg14995160
b
Scale
chr10:
2 kb
hg19
119,000,500
119,001,000
119,001,500
119,002,000
119,002,500
119,003,000
119,003,500
qMSP Amplicons
UCSC Genes (RefSeq, GenBank, CCDS, Rfam, tRNAs & Comparative Genomics)
SLC18A2
Basic Gene Annotation Set from GENCODE Version 19
RP11-501J20.5
Basic Gene Annotation Set from ENCODE/GENCODE Version 7
CpG Islands
CpG Islands (Islands < 300 Bases are Light Green)
GM12878 Methylation 450K Bead Array from ENCODE/HAIB
cg15806304
cg13980799
cg00498305
cg15173134
cg08521987
cg00512279
cg19721867
cg16099210
cg19617377
cg10245915
cg15225091
cg01043119
cg26583753

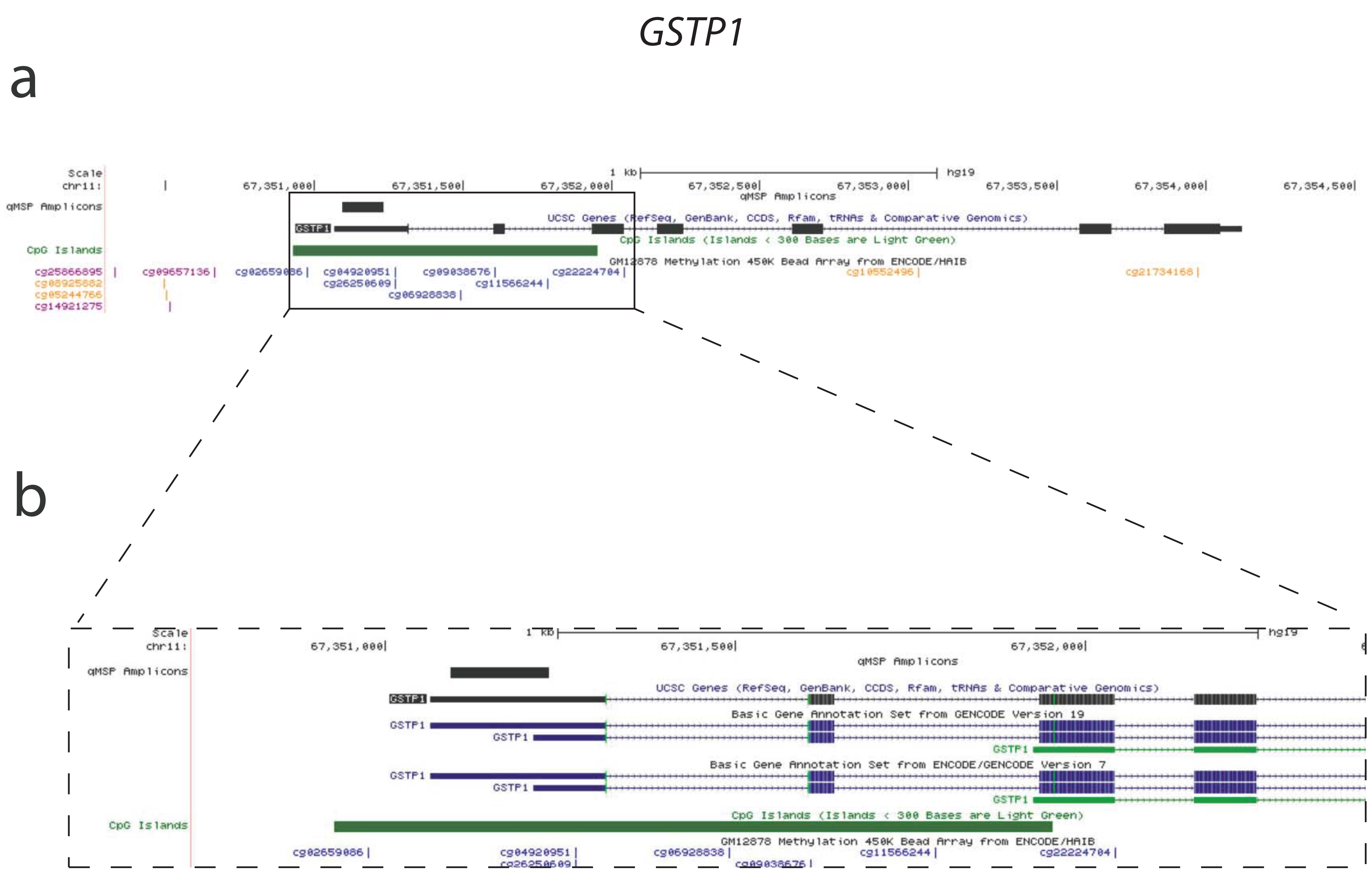

GSTP1
a
Scale
chr11:
1 kb
hg19
67,351,000
67,351,500
67,352,000
67,352,500
67,353,000
67,353,500
67,354,000
67,354,500
qMSP Amplicons
UCSC Genes (RefSeq, GenBank, CCDS, Rfam, tRNAs & Comparative Genomics)
GSTP1
CpG Islands
CpG Islands (Islands < 300 Bases are Light Green)
GM12878 Methylation 450K Bead Array from ENCODE/HAIB
cg25866895
cg08925682
cg05244766
cg14921275
cg09657136
cg02659086
cg04920951
cg26250609
cg09038676
cg06928838
cg11566244
cg22224704
cg10552496
cg21734168
b
Scale
chr11:
1 kb
hg19
67,351,000
67,351,500
67,352,000
qMSP Amplicons
UCSC Genes (RefSeq, GenBank, CCDS, Rfam, tRNAs & Comparative Genomics)
GSTP1
Basic Gene Annotation Set from GENCODE Version 19
GSTP1
GSTP1
GSTP1
Basic Gene Annotation Set from ENCODE/GENCODE Version 7
GSTP1
GSTP1
GSTP1
CpG Islands
CpG Islands (Islands < 300 Bases are Light Green)
GM12878 Methylation 450K Bead Array from ENCODE/HAIB
cg02659086
cg04920951
cg26250609
cg06928838
cg09038676
cg11566244
cg22224704

Supplementary Tables

Supplementary Table S1 –
Multi-gene models and two-gene models where specificity of 100% could be obtained for separation of AN and NM samples in the biopsy set analysed by qMSP

Model	Pearsons χ^2	p	Sensitivity (%)	Specificity (%)	AUC
AOX1xGSTP1	10.42	0.0012	23.1	100.0	0.62
AOX1xHAPLN3	10.42	0.0012	23.1	100.0	0.62
AOX1xSLC18A2	9.13	0.0025	20.5	100.0	0.60
GSTP1xHAPLN3	7.88	0.0050	17.9	100.0	0.59
HAPLN3xSLC18A2	7.88	0.0050	17.9	100.0	0.59
AOX1xGAS6	6.66	0.0099	15.4	100.0	0.58
GSTP1xSLC18A2	5.47	0.0193	12.8	100.0	0.56
GAS6xGSTP1	4.32	0.0376	10.3	100.0	0.55
GAS6xSLC18A2	4.32	0.0376	10.3	100.0	0.55
GAS6xHAPLN3	4.32	0.0376	10.3	100.0	0.55
AOX1xGSTP1xHAPLN3xSLC18A2	14.51	0.0001	30.8	100.0	0.65
AOX1xHAPLN3xSLC18A2	13.11	0.0003	28.2	100.0	0.64

Supplementary Table S2 – Clinicopathological characteristics for all patients in the 450K set

		N	AN	CAN	CAN & AN
**Patients**	n	9	10	19	22
**Samples**	n	9	10	19	CAN: 40, AN:26
**Age, years**	Mean(range)<br>Unknown	65.1 (58 - 80)<br>0	62.3 (47 – 68)<br>0	61.1 (53-72)<br>1	62.5 (49-74)<br>0
**PSA, ng/mL**	Mean(range)<br>Unknown	-<br>-	-<br>-	15.5 (5.0-37.0)<br>1	12.0 (3.0-47.1)<br>0
**Gleason Score**	6<br>7<br>>8<br>Unknown	-<br>-<br>-<br>-	-<br>-<br>-<br>-	1 (5)<br>12 (63)<br>5 (27)<br>1 (5)	9 (41)<br>8 (36)<br>5 (23)<br>0
**pT (%)**	pT2<br>pT3- pT4<br>Unknown	-<br>-<br>-	-<br>-<br>-	10 (53)<br>8 (42)<br>1 (5)	12 (55)<br>10 (45)<br>0
**pN (%)**	pN0<br>pN1<br>pNx	-<br>-<br>-	-<br>-<br>-	1 (5)<br>0<br>18 (95)	15 (68)<br>4 (18)<br>3 (14)

N: non-malignant prostate samples from cystoprostatectomy patients. AN: adjacent normal samples from radical prostatectomies. CAN: malignant samples from radical prostatectomies (one primary tumour was from TURP). pT: Pathological tumour stage. pN: pathological lymph node status.

Supplementary Table S3 - Clinicopathological characteristics for the four patients (PC1-PC4) in the 450K LMD set

**Patient**	**PC1**	**PC2**	**PC3**	**PC4**
**DAN**<br>**PAN**<br>**CAN**	1<br>1<br>2	1<br>1<br>1	1<br>1<br>2	1<br>1<br>1
**Age, years**	61	51	67	49
**PSA, ng/mL**	21.0	28.9	18.1	47.1
**Gleason Score**	7	6	7	8
**Gleason Grades**	4+3, 4+5	3+3	4+4, 4+3	4+3
**pT**	pT3	pT3	pT3	pT3
**pN**	pN1	pN1	pN1	pN1

pT: Pathological tumour stage. pN: pathological lymph node status.

Supplementary Table S4 – Probe IDs and chromosomal positions for probes illustrated in Supplementary Figure S3

Gene	Chromosomal Position	Probe ID	Mean β N	Mean β AN	Mean β CAN
AOX1	**201450506**	**cg04380340**	**0.02**	**0.04**	**0.53**
AOX1	**201450527**	**cg13000082**	**0.05**	**0.06**	**0.55**
AOX1	**201450560**	**cg24569637**	**0.03**	**0.04**	**0.51**
AOX1	**201450575**	**cg08266417**	**0.01**	**0.03**	**0.52**
AOX1	**201450601**	**cg09729613**	**0.03**	**0.03**	**0.48**
AOX1	**201450610**	**cg08952506**	**0.02**	**0.04**	**0.48**
AOX1	**201450633**	**cg02657832**	**0.02**	**0.05**	**0.38**
AOX1	**201450690**	**cg02144933**	**0.02**	**0.03**	**0.45**
AOX1	**201450731**	**cg12627583**	**0.04**	**0.08**	**0.62**
AOX1	**201450743**	**cg13875120**	**0.03**	**0.07**	**0.63**
AOX1	**201451026**	**cg27547291**	**0.27**	**0.30**	**0.53**
AOX1	**201451303**	**cg22953017**	**0.29**	**0.32**	**0.51**
CCDC181	**169395476**	**cg13380669**	**0.90**	**0.83**	**0.74**
CCDC181	**169396635**	**cg00100121**	**0.02**	**0.05**	**0.53**
CCDC181	**169396637**	**cg13958426**	**0.05**	**0.06**	**0.54**
CCDC181	**169396706**	**cg00002719**	**0.03**	**0.06**	**0.56**
CCDC181	**169396712**	**cg08104202**	**0.06**	**0.08**	**0.54**
CCDC181	**169396858**	**cg08047907**	**0.08**	**0.09**	**0.57**
CCDC181	**169396868**	**cg24808280**	**0.10**	**0.09**	**0.52**
GABRE	151142517	cg27049053	0.24	0.26	0.40
GABRE	**151142772**	**cg12204574**	**0.05**	**0.07**	**0.34**
GABRE	**151143125**	**cg08783090**	**0.04**	**0.05**	**0.31**
GABRE	151143213	cg18748981	0.31	0.35	0.42
GABRE	**151143302**	**cg25528646**	**0.05**	**0.05**	**0.39**
GSTP1	**67350976**	**cg02659086**	**0.02**	**0.04**	**0.40**
GSTP1	**67351271**	**cg04920951**	**0.06**	**0.05**	**0.43**
GSTP1	**67351490**	**cg06928838**	**0.07**	**0.09**	**0.58**
GSTP1	**67352041**	**cg22224704**	**0.19**	**0.21**	**0.53**
HAPLN3	89437710	cg16661000	0.04	0.06	0.11
HAPLN3	**89438128**	**cg22138529**	**0.04**	**0.07**	**0.29**
HAPLN3	**89438208**	**cg04829853**	**0.01**	**0.03**	**0.36**
HAPLN3	**89438671**	**cg08348496**	**0.02**	**0.04**	**0.55**
HAPLN3	**89438731**	**cg01367393**	**0.03**	**0.05**	**0.44**
HAPLN3	**89438905**	**cg01647632**	**0.09**	**0.10**	**0.43**
HAPLN3	**89438918**	**cg02071670**	**0.14**	**0.14**	**0.54**
HAPLN3	**89438964**	**cg03628719**	**0.11**	**0.12**	**0.59**
KLF8	56258113	cg14701129	0.84	0.75	0.83
KLF8	**56258387**	**cg01355242**	**0.28**	**0.28**	**0.62**
KLF8	**56258440**	**cg02590710**	**0.23**	**0.28**	**0.67**
KLF8	**56258695**	**cg19505129**	**0.07**	**0.14**	**0.61**
KLF8	**56258808**	**cg22829182**	**0.07**	**0.15**	**0.60**
KLF8	**56258973**	**cg06774787**	**0.14**	**0.18**	**0.53**
KLF8	**56259040**	**cg03610137**	**0.03**	**0.08**	**0.55**
KLF8	**56259094**	**cg03834574**	**0.04**	**0.09**	**0.63**
KLF8	**56259373**	**cg06655100**	**0.09**	**0.15**	**0.64**
KLF8	**56260186**	**cg24268343**	**0.31**	**0.30**	**0.67**
KLF8	56262829	cg25656981	0.96	0.91	0.93

Boldface and **red** probes corresponds to probes marked with black and red triangles, respectively, in Supplementary Figure S3 and illustrate a significant difference in beta values (adjusted p-value < 0.05 in Mann-Whitney U test) between N and CAN samples (**boldface**) or AN and N samples (**red**).

Supplementary Table S4 cont'd – Probe ID and chromosomal position for probes illustrated in Supplementary Figure S3

Gene	Chromosomal Position	Probe ID	Mean β N	Mean β AN	Mean β CAN
MOB3B	27526172	cg14297867	0.33	0.33	0.44
MOB3B	**27528300**	**cg14173147**	**0.07**	**0.09**	**0.53**
MOB3B	**27528432**	**cg21249376**	**0.07**	**0.09**	**0.49**
MOB3B	**27528999**	**cg22262168**	**0.08**	**0.10**	**0.61**
MOB3B	**27529339**	**cg21244846**	**0.07**	**0.07**	**0.58**
MOB3B	27530602	cg14013457	0.90	0.85	0.86
SLC18A2	**119000083**	**cg15806304**	**0.61**	**0.58**	**0.72**
SLC18A2	**119000236**	**cg00498305**	**0.27**	**0.30**	**0.69**
SLC18A2	**119000638**	**cg15173134**	**0.09**	**0.10**	**0.50**
SLC18A2	119000991	cg00512279	0.05	0.06	0.08
SLC18A2	119001066	cg19721867	0.01	0.02	0.02
SLC18A2	119001250	cg16099210	0.04	0.03	0.03
SLC18A2	119001427	cg19617377	0.02	0.06	0.04
SLC18A2	119001478	cg10245915	0.03	0.05	0.07
SLC18A2	119001590	cg15225091	0.17	0.21	0.22

Boldface and **red** probes corresponds to probes marked with black and red triangles, respectively, in Supplementary Figure S3 and illustrate a significant difference in beta values (adjusted p-value < 0.05 in Mann-Whitney U test) between N and CAN samples (**boldface**) or AN and N samples (**red**).

Supplementary Table S5 – Field effects in the 450K LMD subset

Gene	Probe ID	N β Range	AN β Range	PAN (%, n=4)	DAN (%, n=4)	Summary (%, n=4)
AOX1	cg22953017	0.24; 0.38	0.22; 0.42	0 (0)	0 (0)	0 (0)
AOX1	cg13875120	0.01; 0.07	0.05; 0.21	0 (0)	1 (25)	1 (25)
AOX1	cg12627583	0.03; 0.1	0.06; 0.19	0 (0)	0 (0)	0 (0)
AOX1	cg04380340	0.01; 0.04	0.02; 0.32	1 (25)	1 (25)	2 (50)
CCDC181	cg24808280	0.03; 0.17	0.04; 0.5	1 (25)	0 (0)	1 (25)
CCDC181	cg08047907	0.02; 0.12	0.03; 0.58	2 (50)	0 (0)	2 (50)
CCDC181	cg08104202	0.02; 0.09	0.08; 0.45	2 (50)	1 (25)	3 (75)
CCDC181	cg00002719	0; 0.08	0.09; 0.52	2 (50)	1 (25)	3 (75)
CCDC181	cg00100121	0; 0.05	0.04; 0.33	2 (50)	1 (25)	3 (75)
GABRE	cg25528646	0.02; 0.13	0.05; 0.34	1 (25)	0 (0)	1 (25)
GABRE	cg18748981	0.28; 0.37	0.43; 0.52	4 (100)	2 (50)	4 (100)
GABRE	cg12204574	0.02; 0.1	0.04; 0.3	0 (0)	1 (25)	1 (25)
GABRE	cg27049053	0.14; 0.4	0.09; 0.56	1 (25)	0 (0)	1 (25)
GSTP1	cg22224704	0.13; 0.27	0.11; 0.38	2 (50)	1 (25)	3 (75)
GSTP1	cg06928838	0.04; 0.14	0.07; 0.31	1 (25)	0 (0)	1 (25)
GSTP1	cg02659086	0; 0.05	0.02; 0.24	0 (0)	1 (25)	1 (25)
HAPLN3	cg04829853	0; 0.03	0.02; 0.1	0 (0)	0 (0)	0 (0)
HAPLN3	cg03628719	0.03; 0.31	0.08; 0.66	1 (25)	1 (25)	2 (50)
KLF8	cg24268343	0.18; 0.45	0.18; 0.66	1 (25)	0 (0)	1 (25)
KLF8	cg06655100	0.03; 0.23	0.06; 0.25	0 (0)	0 (0)	0 (0)
KLF8	cg03834574	0.02; 0.12	0.03; 0.75	1 (25)	0 (0)	1 (25)
KLF8	cg03610137	0.01; 0.04	0.05; 0.2	1 (25)	2 (50)	2 (50)
KLF8	cg06774787	0.12; 0.17	0.17; 0.51	1 (25)	2 (50)	3 (75)
KLF8	cg22829182	0.04; 0.13	0.1; 0.51	0 (0)	3 (75)	3 (75)
KLF8	cg19505129	0.02; 0.1	0.12; 0.35	4 (100)	2 (50)	4 (100)
KLF8	cg02590710	0.13; 0.29	0.07; 0.75	0 (0)	2 (50)	2 (50)
KLF8	cg01355242	0.17; 0.36	0.03; 0.59	1 (25)	0 (0)	1 (25)
MOB3B	cg21244846	0.02; 0.14	0.06; 0.27	1 (25)	0 (0)	1 (25)
MOB3B	cg22262168	0.03; 0.16	0.08; 0.4	1 (25)	0 (0)	1 (25)
MOB3B	cg21249376	0.01; 0.19	0.02; 0.31	1 (25)	0 (0)	1 (25)
MOB3B	cg14173147	0.03; 0.11	0.04; 0.61	2 (50)	0 (0)	2 (50)
MOB3B	cg14297867	0.21; 0.55	0.09; 0.41	0 (0)	0 (0)	0 (0)
SLC18A2	cg00498305	0.17; 0.36	0.13; 0.66	2 (50)	0 (0)	2 (50)
SLC18A2	cg19617377	0.01; 0.04	0.03; 0.6	1 (25)	1 (25)	2 (50)

N: normal prostate tissue samples from cystoprostatectomy patients from the 450 K set. AN: adjacent normal samples combined, i.e. including DAN and PAN samples from the 450K LMD subset. PAN: Proximal adjacent normal samples from the 450K LMD subset. DAN: Distant adjacent normal samples from the 450K LMD subset. Summary: the total number of patients from the 450K LMD subset with a field effect detected.

Field effect detected in ≤ 10% of patients
Field effect detected in 10-25% of patients
Field effect detected in >25% of patients

Supplementary Methods

The biopsy set:

Bisulphite conversion:

Bisulphite conversion was performed with EZ-96 DNA Methylation-GoldTM Kit (Zymo Research) according to the manufacturer's manual, with an input DNA amount ranging from 240-1000 ng depending on yield during DNA extraction.

Pre-amplification:

A total of 10 ng bisulphite converted DNA was used in a 15 cycle, 15 µl pre-amplification reaction performed in 96-well plates. Temperature conditions for pre-amplification were as follows:

			15 cycles	
	**2 min**	**10 min**	**15 sec.**	**1 min**
AOX1, GABRE, GAS6, GSTP1, HAPLN3, KLF8, MOB3B	50° C	95 °C	95 °C	56 °C
CCDC181,SLC18A2				58 °C

Amplification was performed in a C1000 thermal cycler (Bio-Rad). Mastermix compositions for the 15 µl# pre-amplification reactions:

	**TaqMan* (µl)**	**Template (µl)**	**Primer (pmol)**	**Probe (pmol)**
AOX1	7.5	2	18	3
CCDC181,GABRE, GAS6, HAPLN3, MOB3B, SLC18A2			9	3
GSTP1, KLF8			9	6

#Add water for a total volume of 15 µl, *TaqMan Universal PCR Master mix, No UNG (Life)

qMSP:

For qMSP analysis, 2 µl preamplified DNA + 8 µl mastermix was pipetted into a 384 well plate using the pipetting robot system Biomek 3000 (Beckman Coulter), and qMSP was performed in a 7900HT Fast Real-Time PCR System (Applied Biosystems). All samples were analysed in triplicates and standard curves based on serially diluted methylated DNA were included on each plate. Two control samples were included, one no template control (NTC) containing water and one unmethylated control containing whole genome amplified DNA (WGA). Temperature conditions for qMSP were as for the pre-amplification; however 50 cycles were performed instead of 15. AluC4 was not pre-amplified and qMSP conditions were:

		45 cycles	
	**10 min**	**15 sec.**	**1 min**
AluC4	95 °C	95 °C	60 °C

Furthermore, mastermix compositions for the 10 µl¤ qMSP reactions were:

<table>
<tr><th></th><th>TaqMan* (µl)</th><th>Template (µl)</th><th>Primer (pmol)</th><th>Probe (pmol)</th></tr>
<tr><td>AOX1</td><td rowspan="4">5</td><td rowspan="4">2</td><td>12</td><td>2</td></tr>
<tr><td>CCDC181, GABRE, GAS6, HAPLN3, MOB3B, SLC18A2</td><td>6</td><td>2</td></tr>
<tr><td>GSTP1, KLF8</td><td>6</td><td>4</td></tr>
<tr><td>AluC4</td><td>5</td><td>2</td></tr>
</table>

¤ Add water for a total volume of 10 µl, *TaqMan Universal PCR Master mix, No UNG (Life)

Primers and probes for pre-amplification and qMSP:

Assay	Sense/Forward	Antisense/reverse	Probe
AOX1	TTTCGTAATAGCGGTTTTGT	CAAAACAATCCCTAAAAACG	HEX_TCGTATTTTTATTTTTGTTTTCGGG_BHQ1
CCDC181	ATTTGCGTAGGCGTATGTAA	CGTAAATTCCTTAATCGTCCC	HEX_TCGGTGTTTGCGAAGGGTTAG_BHQ1
GAS6	GGTTTCGTTTTGTTAGGTGT	ATTCCTAACCGAAATACCG	FAM_GCGGGTGTTTTTAGGAGTTCG_BHQ1
GSTP1	AGTTGCGCGGCGATTTC	GCCCCAATACTAAATCACGAC	FAM_CGGTCGACGTTCGGGGTGTAGCG_BHQ1
HAPLN3	CTTTTCGTAGTGTTCGGTTTAC	GAATTCCTCCCTTACCGC	HEX_TCGGATTTTGTTCGGGAGGT_BHQ1
KLF8	TTTAGTAGGGTTAGAGAAAGACG	AACTACCCCACGAAATACCT	FAM_AGGTTTTGATTCGCGTGGGGC_BHQ1
MOB3B	CGTGAGGTATCGTTAGTTTCG	TCAAAAAAACAACGCTTAAACG	TAMRA_CGATTTGGTTGACGGTGCG_BHQ2
SLC18A2	TTTAAGGTATTCGGTTACGCGT	TCGCTACGCAAAAAAAAACTACCG	FAM_TTCGGGGAAGAGGCGCGGTCG_BHQ1
GABRE	GATGTTTAGGAGGATTGGAAGA	CTCCGCGCAAATAATCG	HEX_ATATTTTCGCGGAGATCGGC_BHQ1
AluC4[1]	GGTTAGGTATAGTGGTTTATATTTGTA ATTTTAGTA	ATTAACTAAACTAATCTTAAACTC CTAACCTCA	FAM_CCTACCTTAACCTCCC_MGBNFQ

1. Weisenberger, D. J. *et al.* Analysis of repetitive element DNA methylation by MethyLight. *Nucleic Acids Res.* **33,** 6823–36 (2005).
